# Supplementary material for: Stepwise introduction of three different transition metals in metallo-supramolecular polymer for quad-color electrochromism
Source: Commun Chem. 2021 May 3;4:56. doi: 10.1038/s42004-021-00495-1 (PMC9814570; doi:10.1038/s42004-021-00495-1)
Supplement: Supplementary file 1 — Supplementary Information [file 42004_2021_495_MOESM1_ESM.pdf]

Supporting Information

Stepwise Introduction of Three Different Transition Metals in Metallo-Supramolecular Polymer for Quad-Color Electrochromism

Manas Kumar Bera[1], Yoshikazu Ninomiya[1], and Masayoshi Higuchi[1]*

[1] Electronic Functional Macromolecules Group, Research Center for Functional Materials, National Institute for Materials Science (NIMS), 1-1 Namiki, Tsukuba, Ibaraki 305-0044, Japan

Email: HIGUCHI.Masayoshi@nims.go.jp

Table of Contents

Contents **Page no.**

1. Supplementary Methods

1.1. General information 3

1.2. Synthetic strategy 4

1.3. Syntheses and characterization of TOsT, TOsRuBr, and TOsRuT (NMR, ESI-MS, UV-vis., and CV) 5-22

1.4. Synthesis and characterization of polyOsRuFe (NMR, FT-IR, TGA, UV-vis., and CV) 23-30

1.5. Synthesis and characterization of polyOsRuCo (NMR, XPS, UV-vis., and CV) 31-34

1.6. Synthesis and characterization of polyOsRuZn (NMR, XPS, UV-vis., and CV) 35-38

1.7. Synthesis and characterization of polyOsRuFe-A (A: BF_4, Cl, PF_6, OAc) (UV-vis., CV, and solubility) 39-47

1.8. Spectroelectrochemical study of polyOsRuFe-OAc film on ITO/glass 48-49

2. Supplementary References 50

1. Supplementary Methods

1.1. General information

Compound 3: 4'-(4-(4,4,5,5-tetramethyl-1,3,2-dioxaborolan-2-yl)phenyl)-2,2':6',2''-terpyridine,[1] BrtpyOsCl_3,[2] BrtpyRuCl_3 (3**)**,[2] Compound 2,[3] and TOsT[3] were prepared according to the reported literatures.

<u>*Assignment of ^{1}H NMR for 2,2':6',2''-terpyridine unit:*</u>

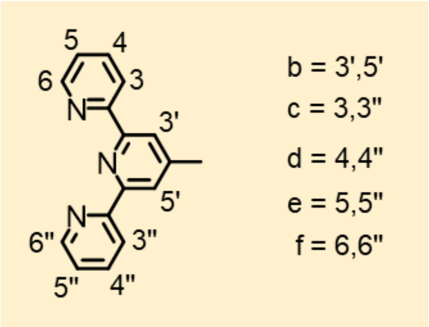

1.2. Synthetic strategy

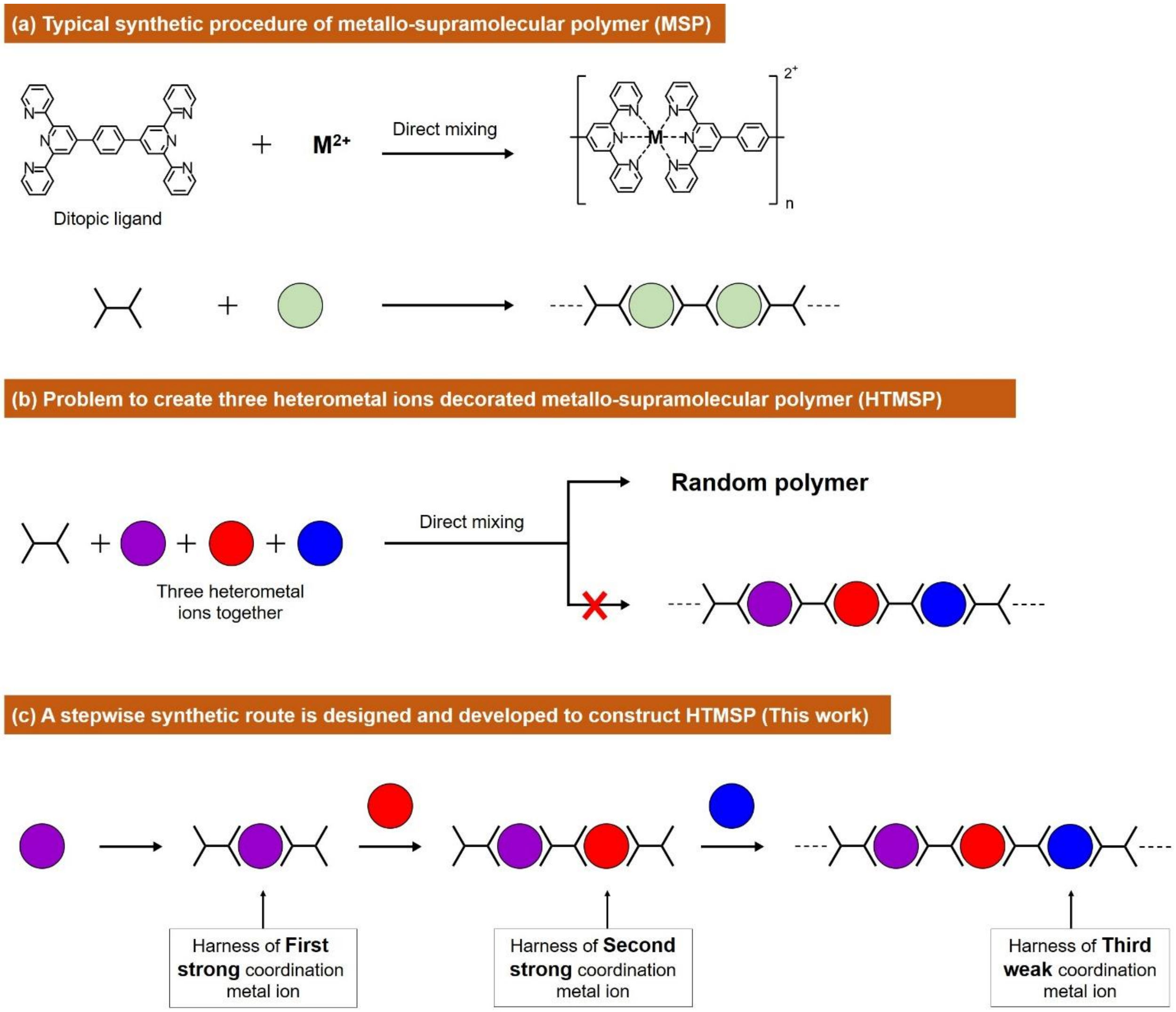

Supplementary Figure 1. Schematic presentation of the synthesis of heterotrimetallic supramolecular polymer (HTMSP). **a** Typical synthetic procedure for metallo-supramolecular polymer (MSP) by coordination-driven self-assembly of a ditopic ligand and one metal ion. **b** Problem to construct HTMSP through direct mixing of a ditopic ligand and three heterometal ions, and **c** Synthetic route developed in this study to construct HTMSP.

1.3. Syntheses and characterization of TOsT, TOsRuBr, and TOsRuT

During the synthesis of TOsRuT with Cl^- counteranions, initially, introduction of Os(II) and Ru(II) was targeted in step wise manner using $BrtpyOsCl_3$ and $BrtpyRuCl_3$ in two ways as shown below in Supplementary Figure 2. But both approaches were unsuccessful (Approach-1 and Approach-2 in Supplementary Figure 2) to obtain TOsRuT. The reason behind this failure may be due to (i) the temperature difference for binding of Os(II) and Ru(II) with 2,2':6',2"-terpyridine (tpy) [the tpy generally bind Os(II) at high temperature compared to Ru(II)] and/or (ii) undesired side product formation associated with $BrtpyOsCl_3$, which produces mixture of products with very low yield of desired product that creates difficulty to isolate desired product by column chromatography.[4-6] Therefore, we have designed and developed a step wise synthetic route for precise synthesis of TOsRuT and to get significant yield of product in each step (Supplementary Figure 3). In this designed synthetic route, we have avoided the use of $BrtpyOsCl_3$ to prevent undesired side products formation.

Our target was to synthesize TOsRuT with Cl^- as counteranions:

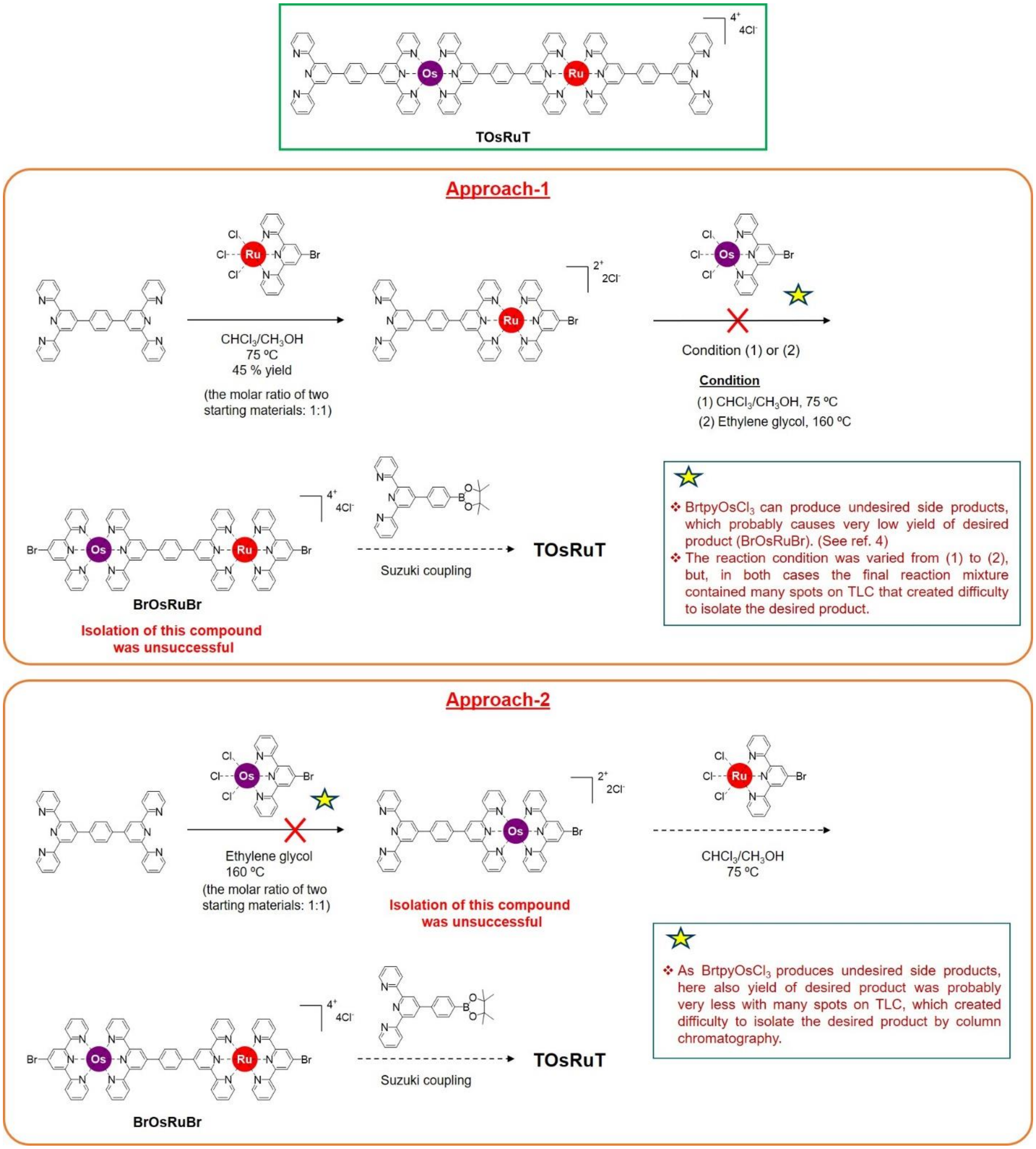

Supplementary Figure 2. Synthetic approaches that were unsuccessful to prepare TOsRuT.

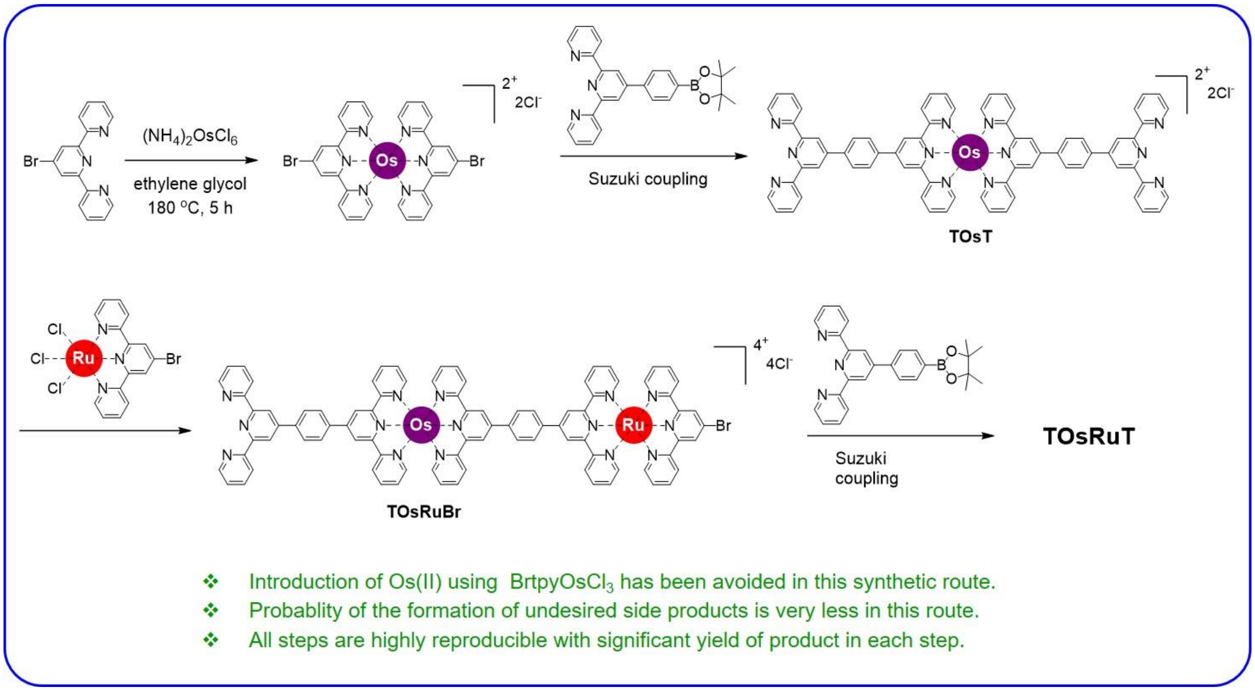

Supplementary Figure 3. Synthetic route that was designed and developed to prepare TOsRuT.

Synthesis of TOsT[3]

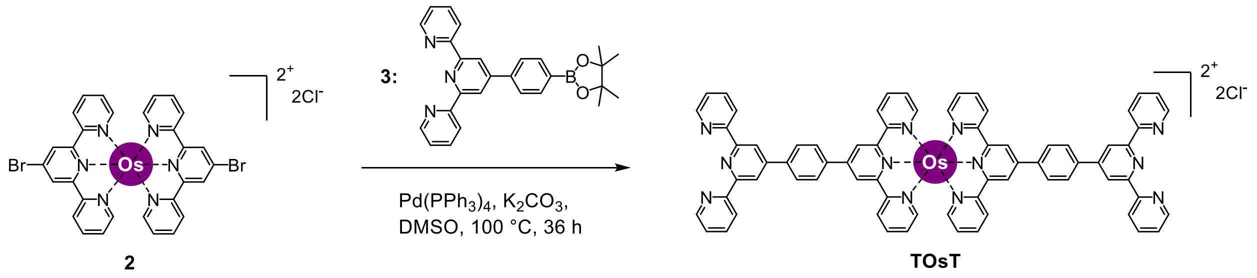

Compound 2 (407.3 mg, 0.46 mmol) and compound 3 (1.001 g, 2.30 mmol), K_2CO_3 (160.4 mg, 1.16 mmol), and $Pd(PPh_3)_4$ (60 mg, 0.051 mmol) were taken in a 100 mL two-neck round-bottom flask. Then, 40 mL DMSO was added to it under a nitrogen atmosphere and heated at 100 °C for 36 h. After removing DMSO, the resulting solid was purified by column chromatography (SiO_2) eluting with CH_2Cl_2/CH_3OH (4:1, v/v) to afford TOsT as a black solid (450.6 mg, 73% yield). **^{1}H NMR** (CD_2Cl_2/CD_3OD [1:1, v/v], 400 MHz, ppm): δ 9.27 (s, 4H), 8.87-8.82 (m, 8H), 8.77-8.74 (m, 8H), 8.44-8.35 (m, 8H), 8.04 (t, 4H). 7.92 (t, 4H), 7.51 (t, 4H), 7.38 (d, 4H), 7.25 (t, 4H). **ESI-MS (m/z):** (*m/z*): 636.1090 $[TOsT\text{-}2Cl^-]^{2+}$ (calcd *m/z* = 636.1867). **UV-vis** [5 × 10^{-6} M in CH_2Cl_2/CH_3OH (1:1, v/v)]: 314 nm (for π-π* transition), 493 nm [singlet MLCT for <tpy-Os(II)-tpy> connectivity], and 669 nm [triplet MLCT for <tpy-Os(II)-tpy> connectivity].

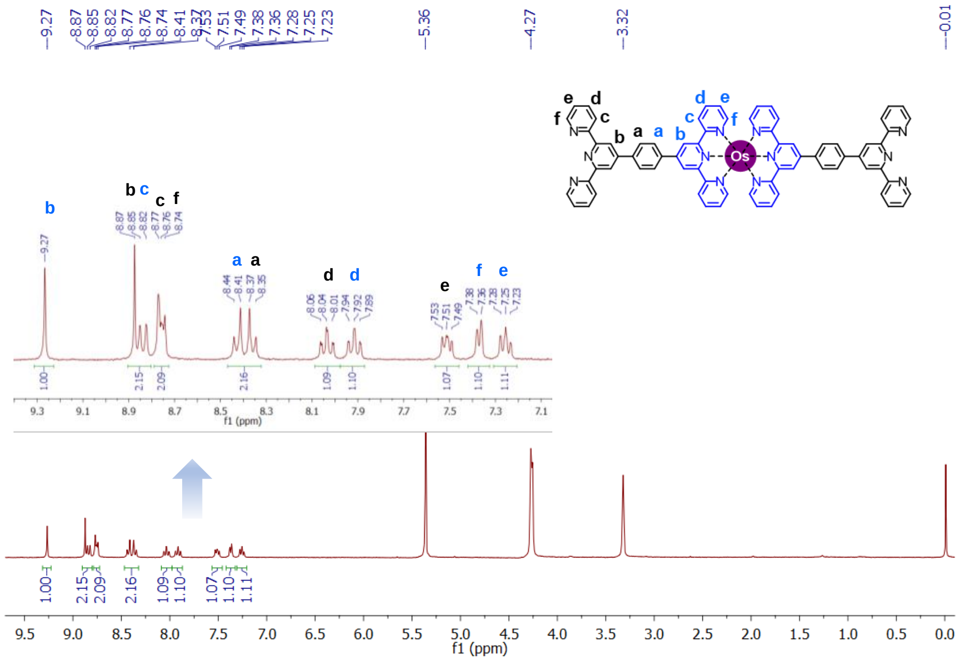

Supplementary Figure 4. ^{1}H NMR spectrum of TOsT in CD_2Cl_2/CD_3OD, 1:1 (v/v).

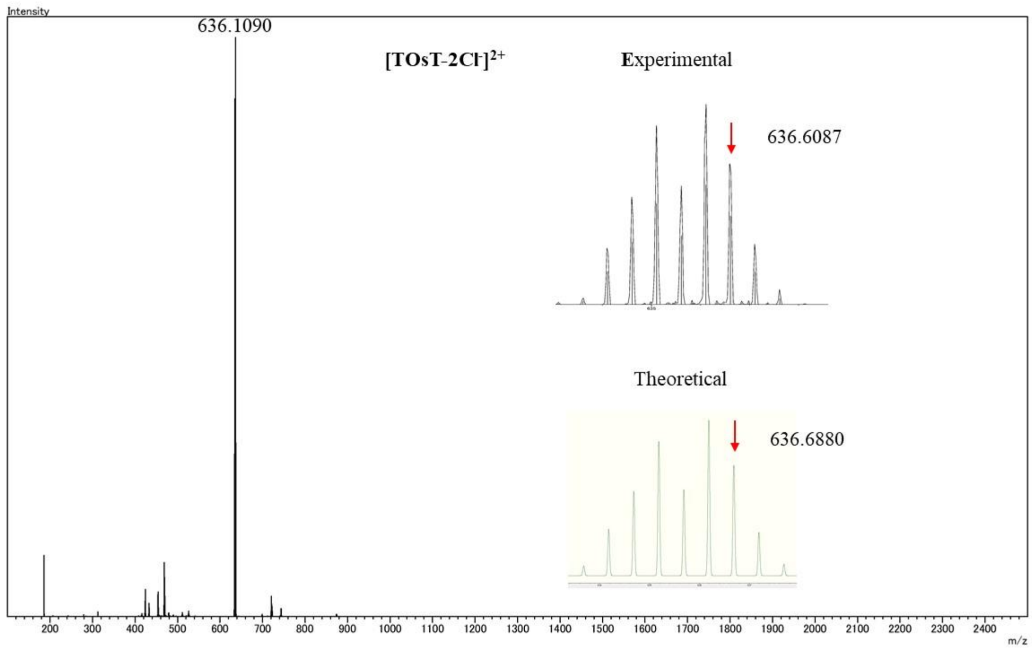

Supplementary Figure 5. ESI mass spectrum of TOsT.

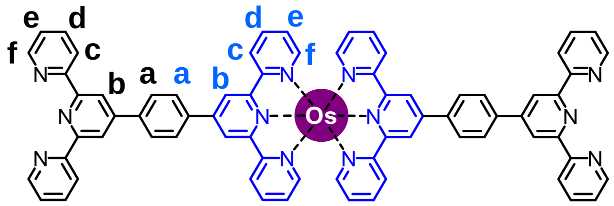

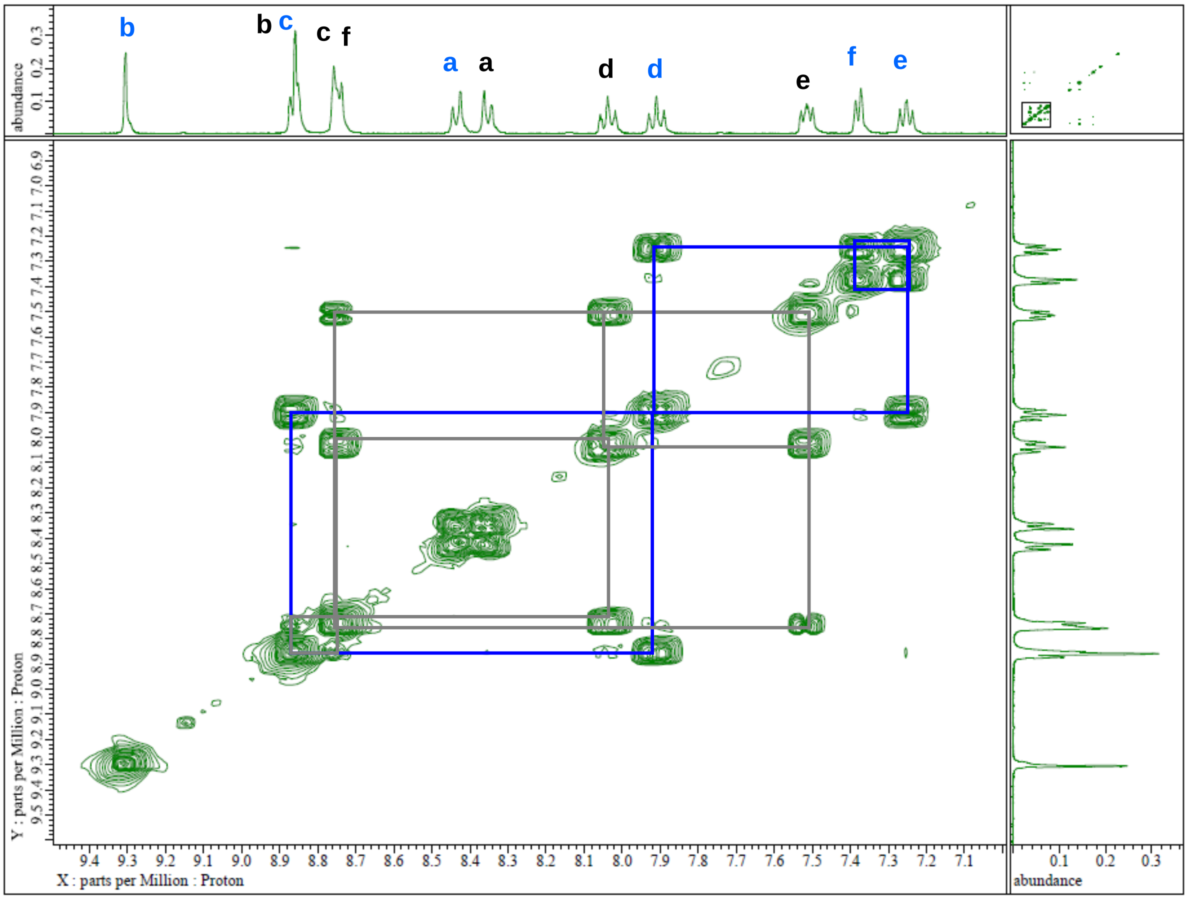

Supplementary Figure 6. COSY NMR spectrum of TOsT in CD_2Cl_2/CD_3OD, 1:1 (v/v).

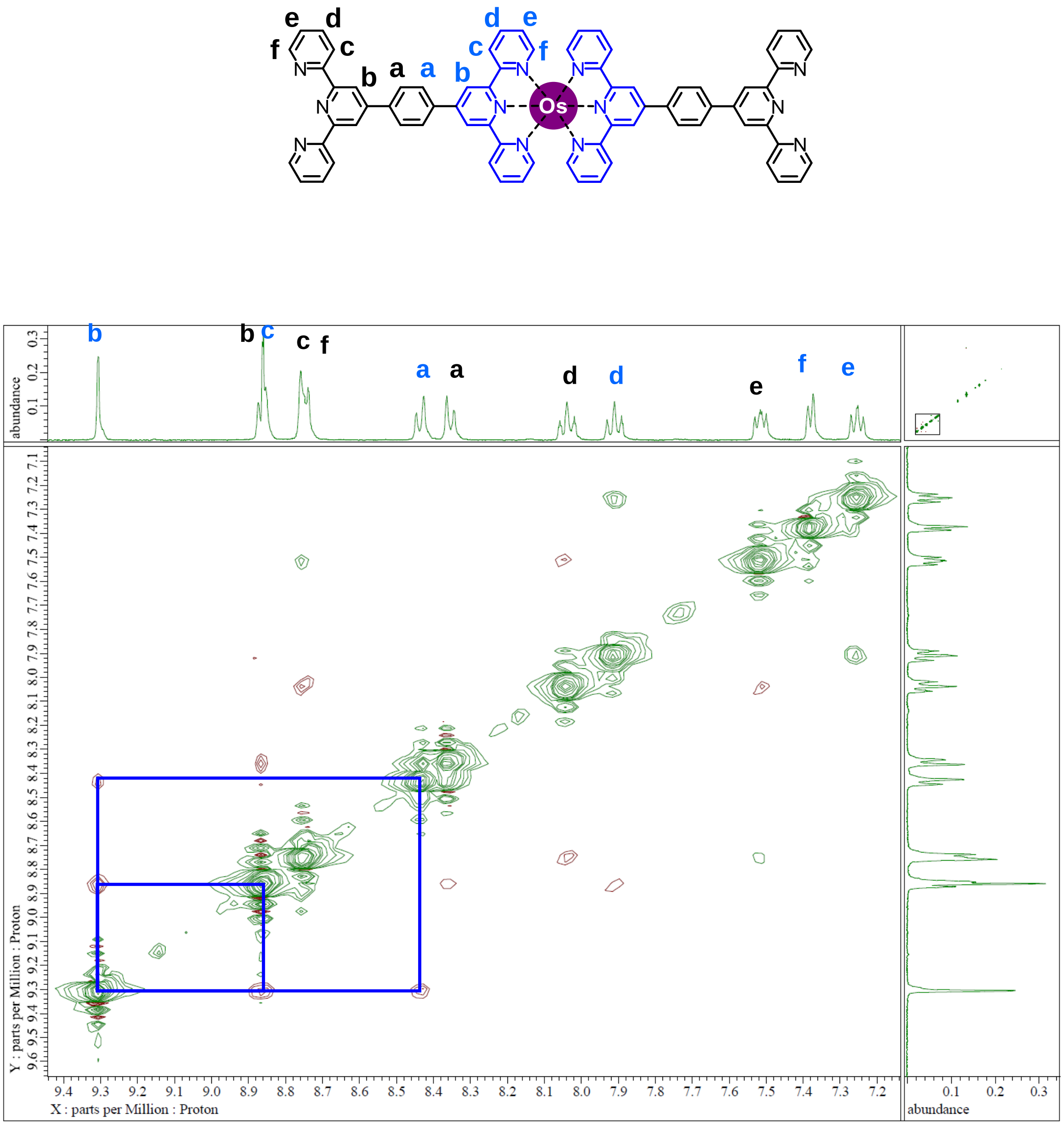

Supplementary Figure 7. NOESY NMR spectrum of TOsRuBr in CD_2Cl_2/CD_3OD, 1:1 (v/v).

Synthesis of TOsRuBr

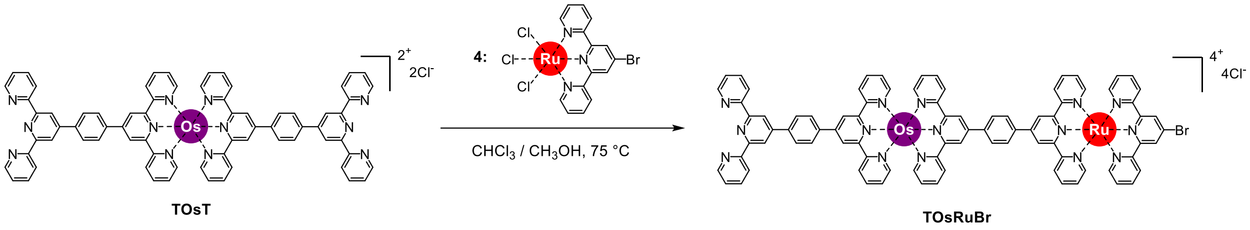

In a 100 mL two-neck round-bottom flask, TOsT (100 mg, 74.5 µmol) and compound 4 (38.7 mg, 74.5 µmol) were dissolved in 60 mL solvent mixture of $CHCl_3$ and CH_3OH (1:1, v/v) and stirred for 30 min. Then, ten drops 4-ethylmorpholine was added to the reaction mixture and heated at 75 °C for 15 h. The solvent was removed under reduced pressure to give a deep brown solid, which was purified by column chromatography (Al_2O_3) eluting with CH_2Cl_2 /CH_3OH, 9:1 (v/v) to collect first fraction as desired product (a second fraction of undesired product was appeared, which was probably the product of both side attachment of compound 4 with TOsT) to afford TOsRuBr, as brown powder (60.2 mg, 44% yield). **^{1}H NMR** (CD_2Cl_2/CD_3OD [1:1, v/v], 400 MHz, ppm): δ 9.52 (s, 2H), 9.48 (s, 2H), 9.34 (s, 2H), 9.22 (s, 2H), 9.06 (d, 4H), 8.89-8.88 (m, 4H), 8.78-8.70 (m, 10H), 8.46 (d, 2H), 8.39 (d, 2H), 8.10-8.02 (m, 6H), 7.98-7.92 (q, 4H), 7.57-7.52 (m, 4H), 7.45 (d, 4H), 7.40-7.28 (m, 10H). **^{13}C NMR** (CD_2Cl_2/CD_3OD [1:1, v/v], 400 MHz, ppm): δ 160.94, 160.85, 159.09, 157.96, 157.00, 156.78, 156.48, 156.17, 156.09, 153.08, 153.01, 152.93, 149.82, 139.47, 139.35, 139.19, 139.14, 138.61, 132.39, 130.33, 129.80, 129.20, 129.00, 128.80, 128.05, 126.35, 126.19, 125.33, 122.81, 122.71, 121.36, 121.14, 119.67. **ESI-MS (m/z):** (*m/z*): 421.2714 [TOsRuBr-4Cl$^-$]$^{4+}$ (calcd *m/z* = 421.3205).

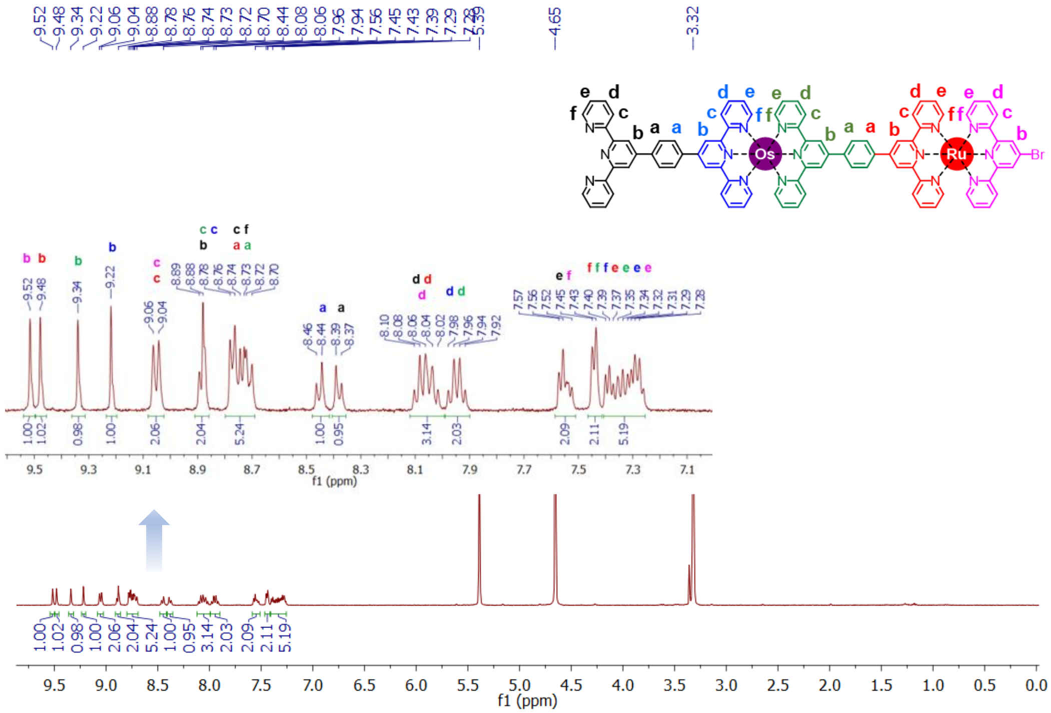

Supplementary Figure 8. ^{1}H NMR spectrum of TOsRuBr in CD_2Cl_2/CD_3OD, 1:1 (v/v).

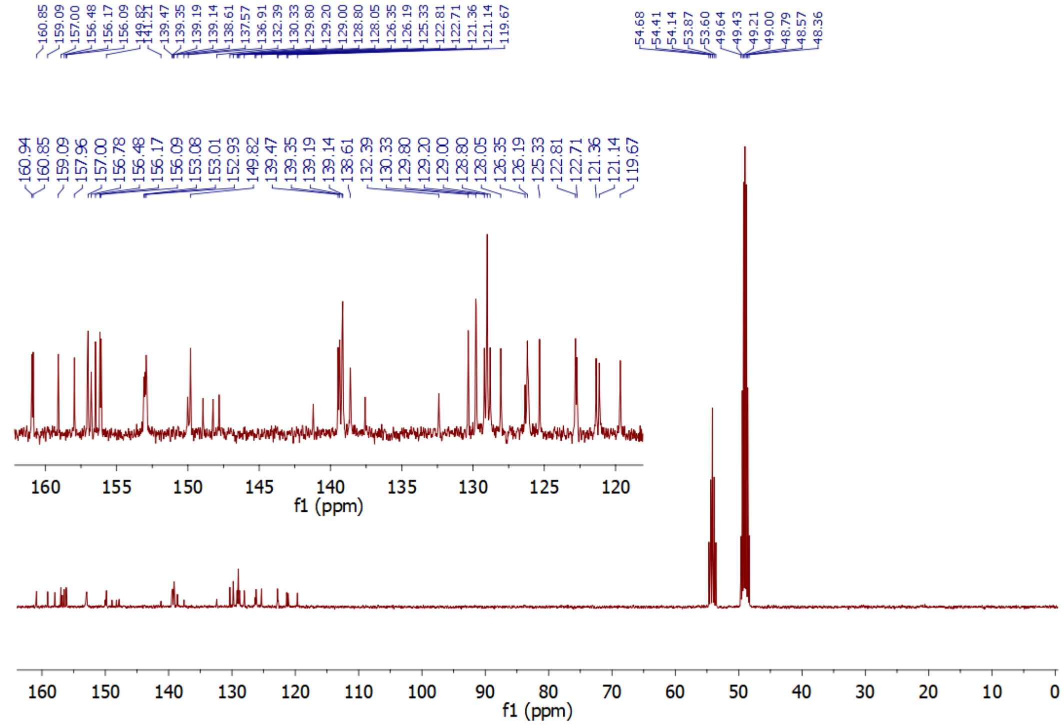

Supplementary Figure 9. ^{13}C NMR spectrum of TOsRuBr in CD_2Cl_2/CD_3OD, 1:1 (v/v).

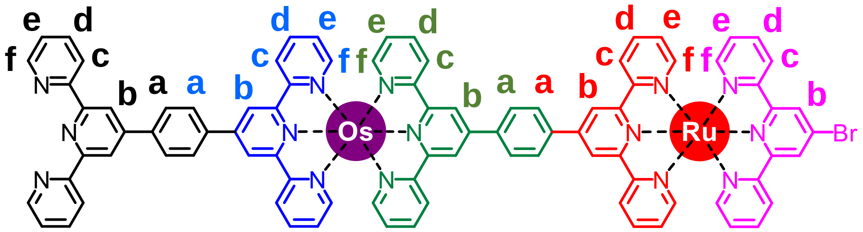

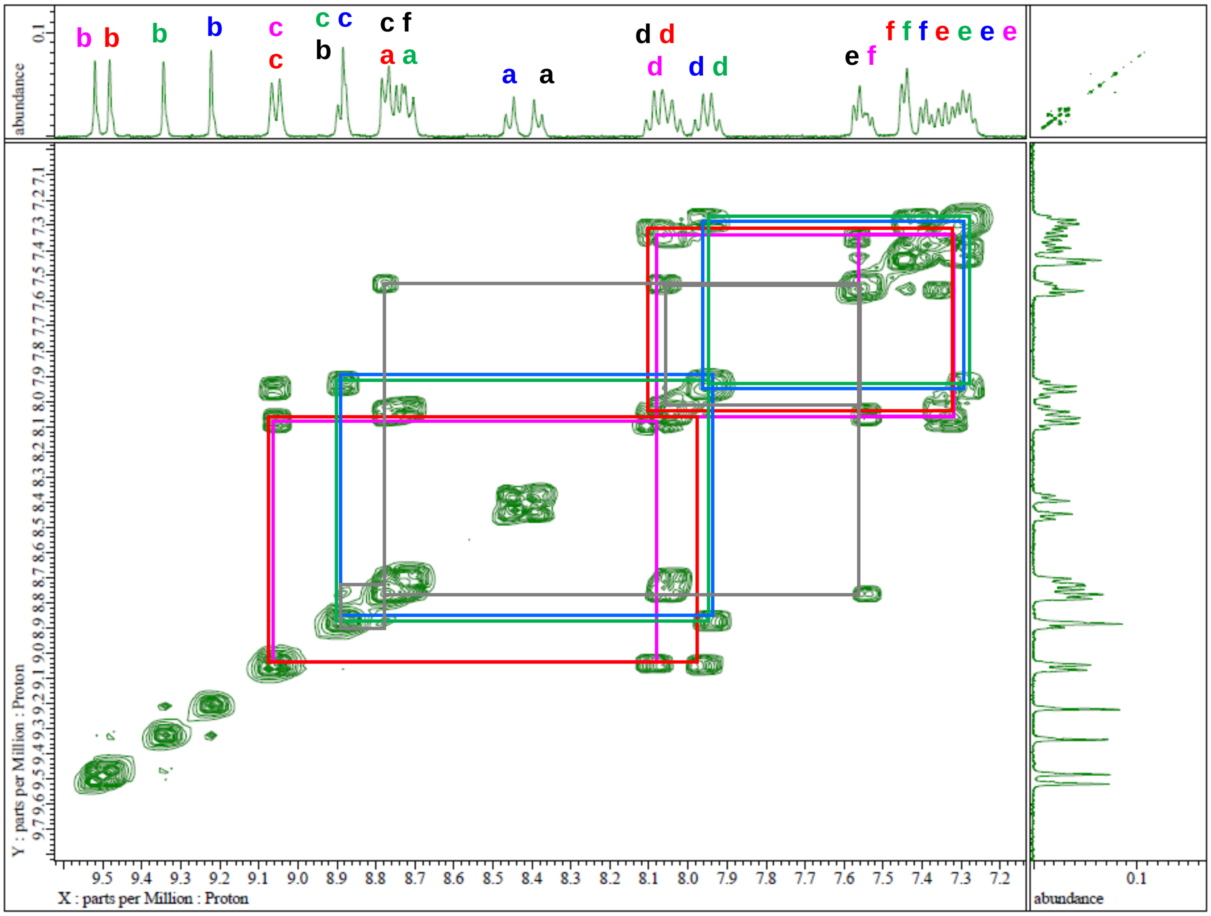

Supplementary Figure 10. COSY NMR spectrum of TOsRuBr in CD_2Cl_2/CD_3OD, 1:1 (v/v).

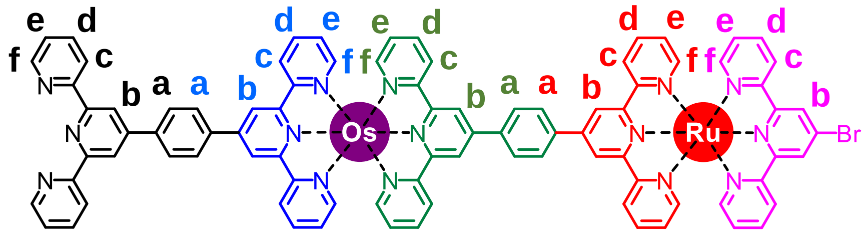

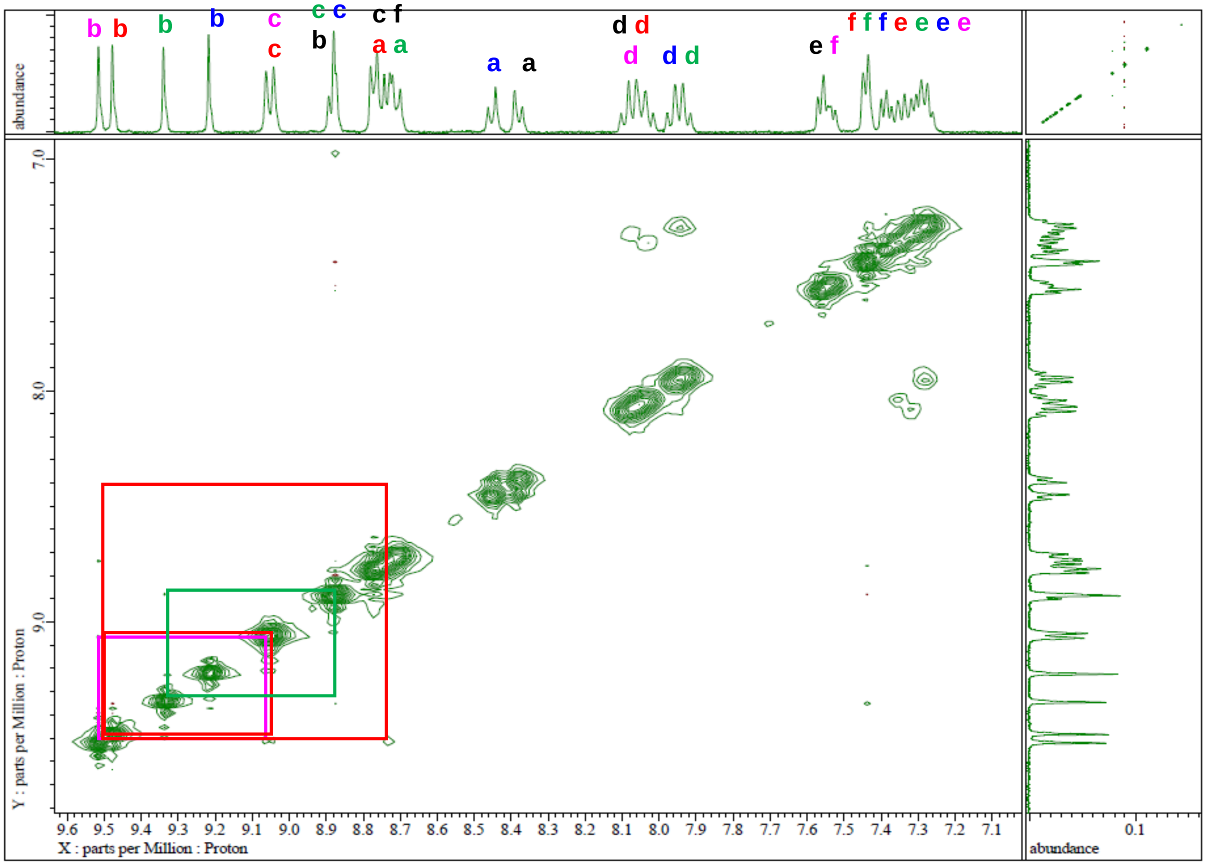

Supplementary Figure 11. NOESY NMR spectrum of TOsRuBr in CD_2Cl_2/CD_3OD, 1:1 (v/v).

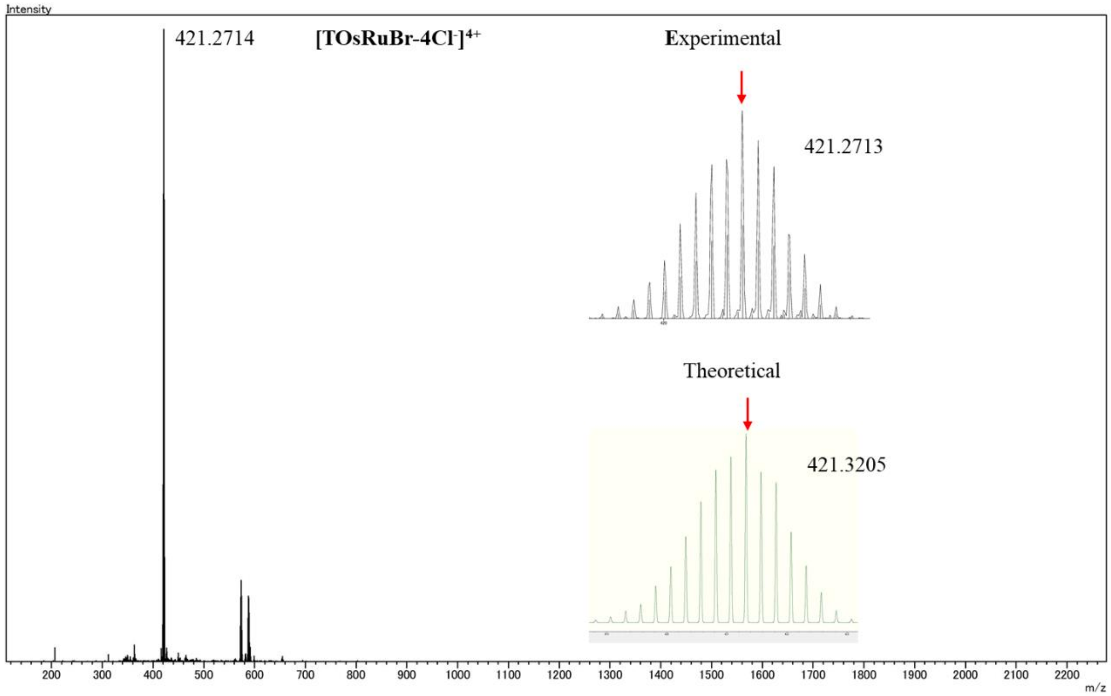

Supplementary Figure 12. ESI mass spectrum of TOsRuBr.

Synthesis of TOsRuT

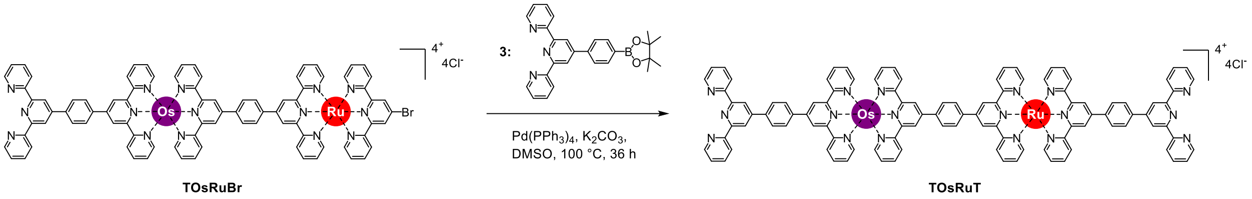

In a 50 mL two-neck round-bottom flask TOsRuBr (100 mg, 54.6 µmol), compound 3 (142.8 mg, 0.32 mmol), K_2CO_3 (19.3 mg, 0.14 mmol), and Pd(PPh_3)$_4$ (9.4 mg, 8.1 µmol, 15%) were dissolved in 20 mL anhydrous DMSO under a nitrogen atmosphere. The reaction mixture was heated at 85 °C for 24 h. The DMSO was removed under vacuum and the residue was purified by column chromatography (Al_2O_3) eluting with CH_2Cl_2 /CH_3OH, 9:1 (v/v) first and then, CH_2Cl_2 /CH_3OH, 4:1 (v/v) to afford TOsRuT, as deep brown solid (76.4 mg, 68% yield). **^{1}H NMR** (CD_2Cl_2/CD_3OD [1:1, v/v], 400 MHz, ppm) δ 9.53 (d, 4H), 9.35 (d, 4H), 9.10-9.07 (m, 4H), 8.93-8.89 (m, 8H), 8.81-8.74 (m, 12H), 8.52-8.47 (q, 4H), 8.40 (d, 4H), 8.13-8.06 (m, 8H), 7.98-7.92 (q, 4H), 7.59-7.58 (m, 6H), 7.54 (d, 2H), 7.47 (d, 2H), 7.41-7.28 (m, 10H). **^{13}C NMR** (CD_2Cl_2/CD_3OD [1:1, v/v], 400 MHz, ppm): δ 160.96, 160.87, 159.21, 159.12, 157.10, 157.06, 156.78, 156.59, 156.54, 156.20, 156.12, 153.02, 152.90, 150.09, 150.02, 149.82, 141.27, 139.40, 139.35, 139.22, 139.14, 138.63, 130.34, 129.77, 129.43, 129.28, 129.03, 128.84, 126.14, 125.36, 122.84, 122.66, 122.41, 121.36, 121.13, 119.66. **ESI-MS (m/z):** (*m/z*): 478.5632 [TOsRuT-4Cl^-]$^{4+}$ (calcd *m/z* = 478.6221). **UV-Vis** [5 × 10^{-6} M in CH_2Cl_2/CH_3OH (1:1, v/v)]: 311 nm (for π-π* transition), 500 nm [MLCT for <tpy-Ru(II)-tpy> connectivity + singlet MLCT for <tpy-Os(II)-tpy> connectivity], and 671 nm [triplet MLCT for <tpy-Os(II)-tpy> connectivity]. **CV** ($E_{1/2}$): 0.63 and 0.99 V for Os(II)/Os(III) and Ru(II)/Ru(III) redox pair, respectively.

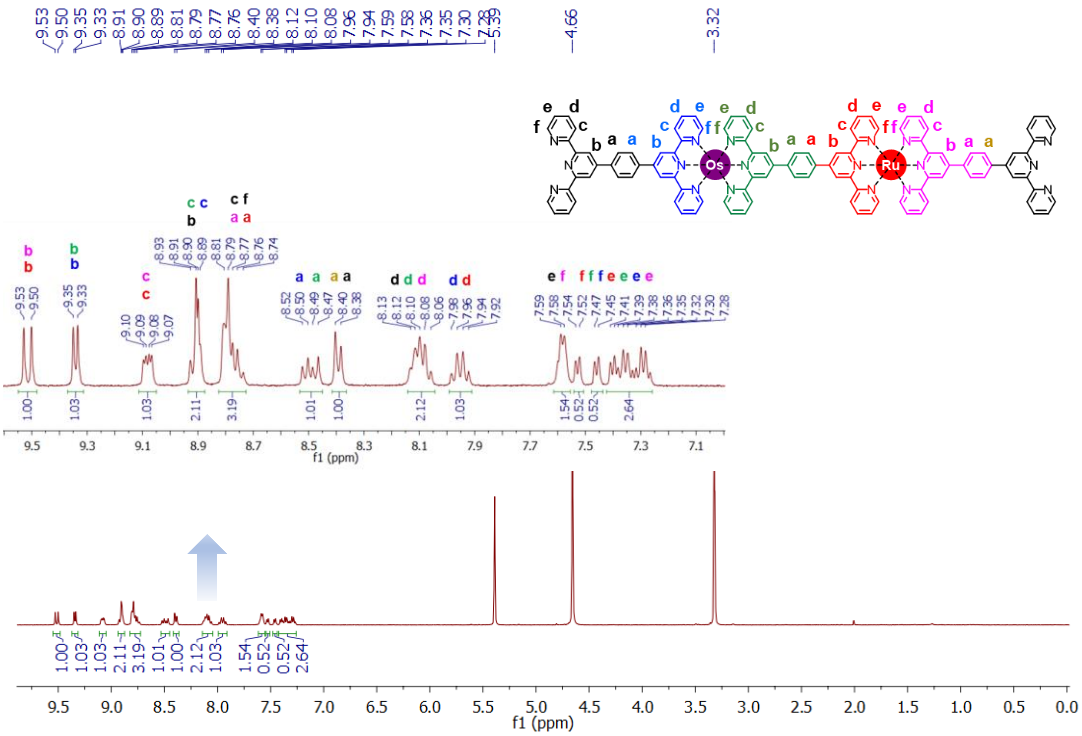

Supplementary Figure 13. ^{1}H NMR spectrum of TOsRuT in CD_2Cl_2/CD_3OD, 1:1 (v/v).

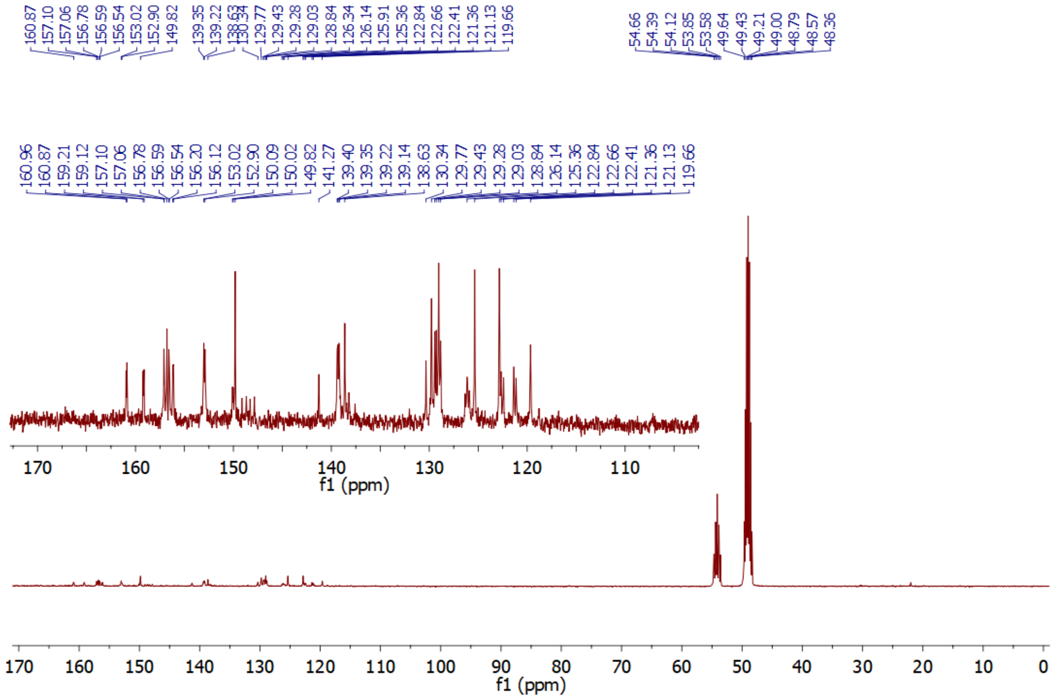

Supplementary Figure 14. ^{13}C NMR spectrum of TOsRuT in CD_2Cl_2/CD_3OD, 1:1 (v/v).

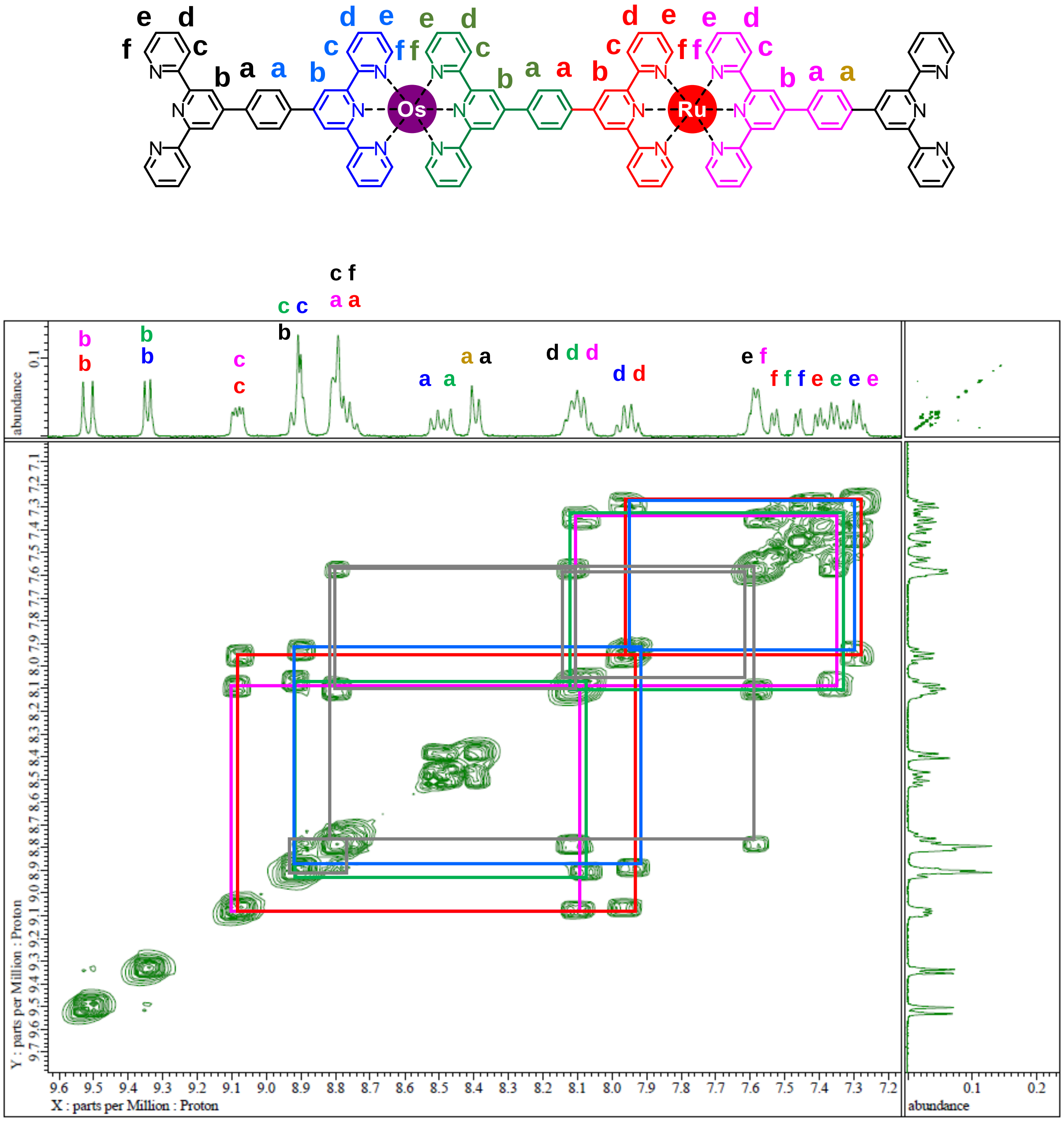

Supplementary Figure 15. COSY NMR spectrum of TOsRuT in CD_2Cl_2/CD_3OD, 1:1 (v/v).

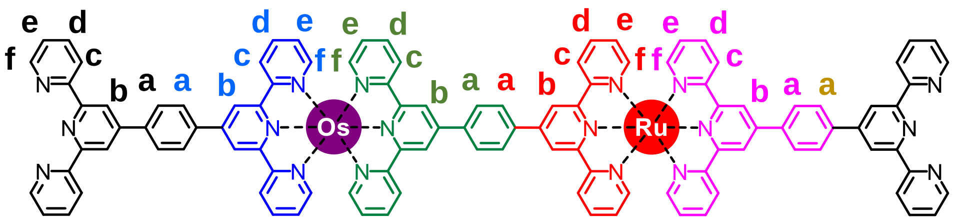

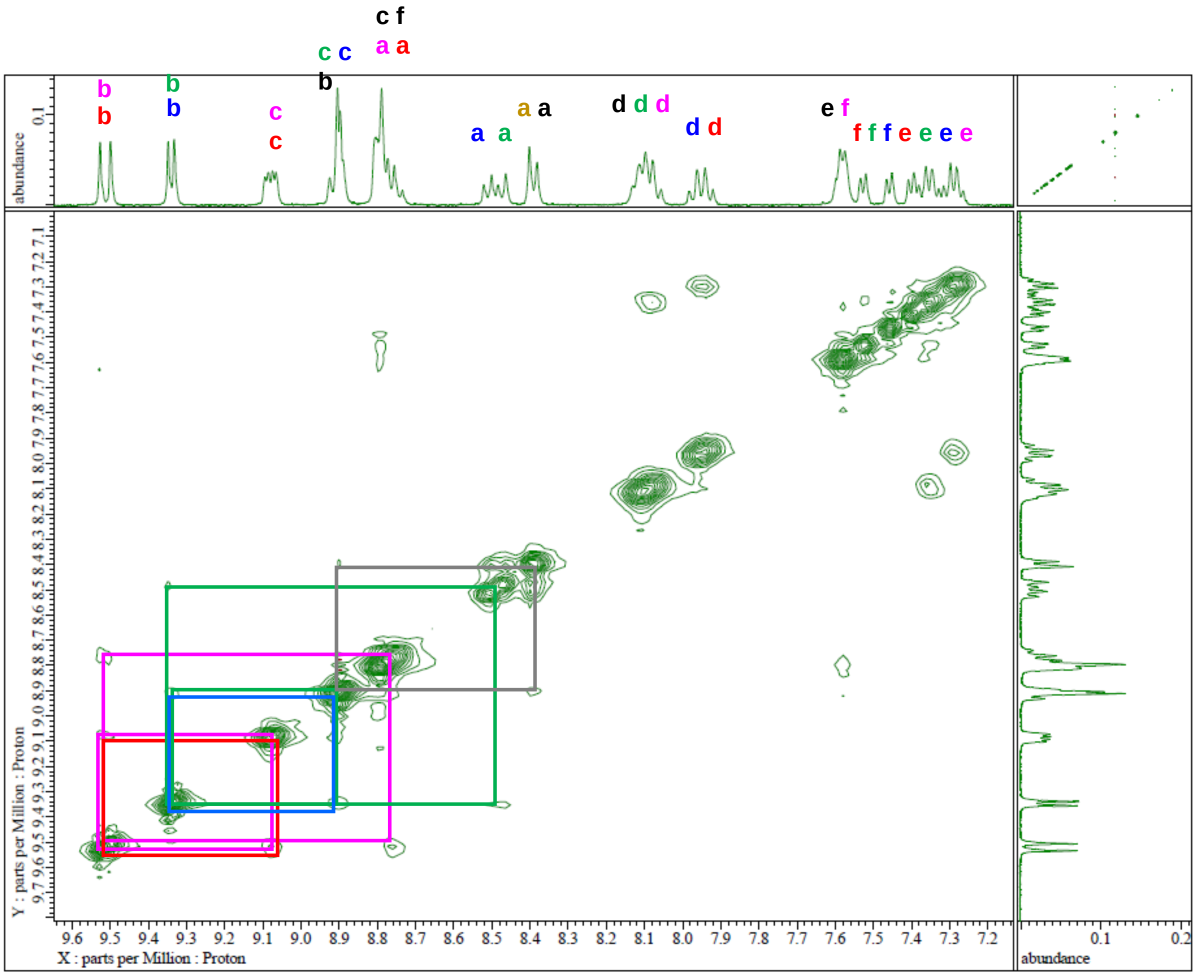

Supplementary Figure 16. NOESY NMR spectrum of TOsRuT in CD_2Cl_2/CD_3OD, 1:1 (v/v).

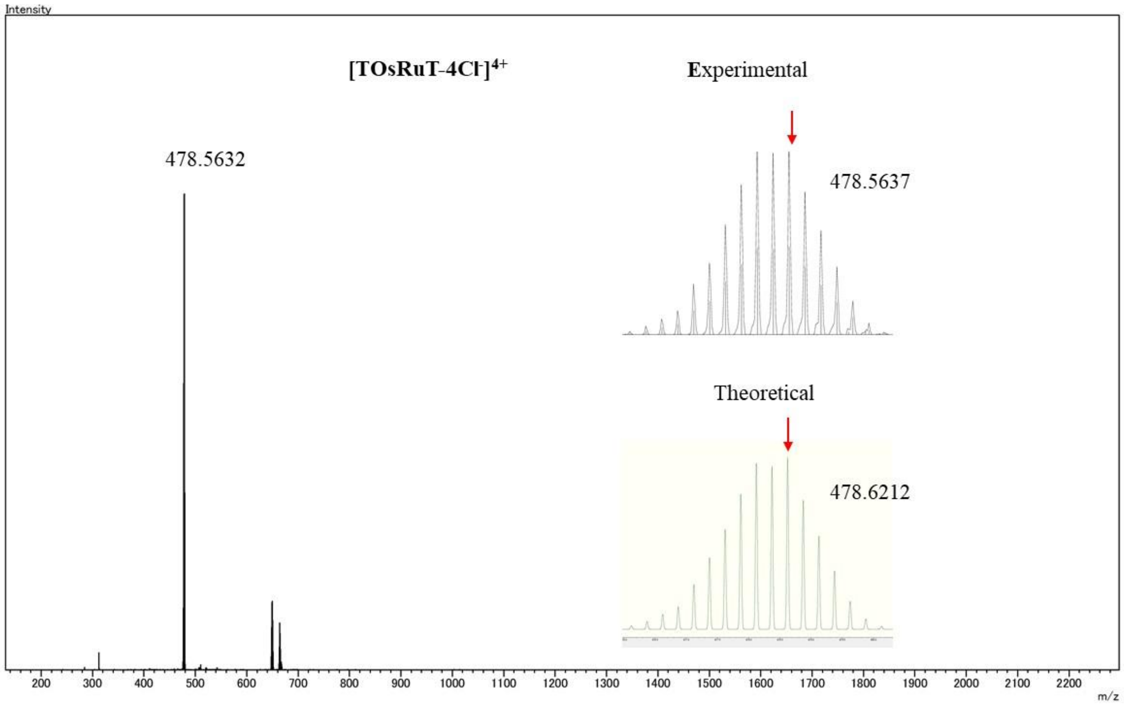

Supplementary Figure 17. ESI mass spectrum of TOsRuT.

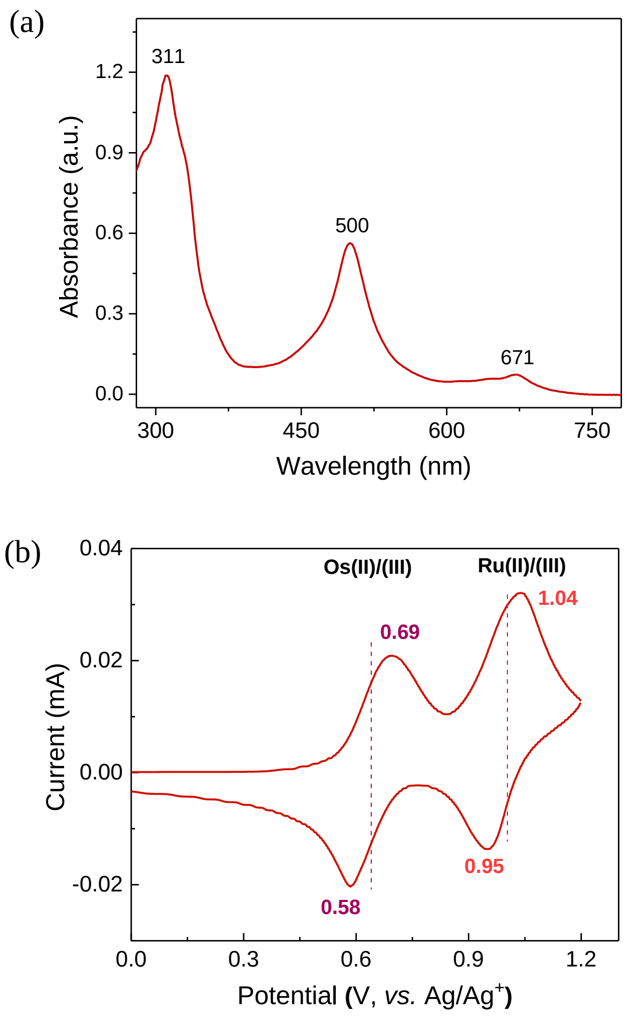

Supplementary Figure 18. a UV-vis spectrum [5 × 10^{-6} M in CH_2Cl_2/CH_3OH, 1:1 (v/v)] and **b** cyclic voltammogram (in three electrode system; glassy carbon as working electrode, platinum wire as counter electrode, and Ag/Ag^+ as reference electrode, electrolyte: 0.1 M $LiClO_4$ in CH_3CN, scan rate 50 mV/s) of TOsRuT. The TOsRuT was dissolved in CH_2Cl_2/CH_3OH, 1:1 (v/v) and drop casted on the glassy carbon electrode.

1.4. Synthesis and characterization of polyOsRuFe

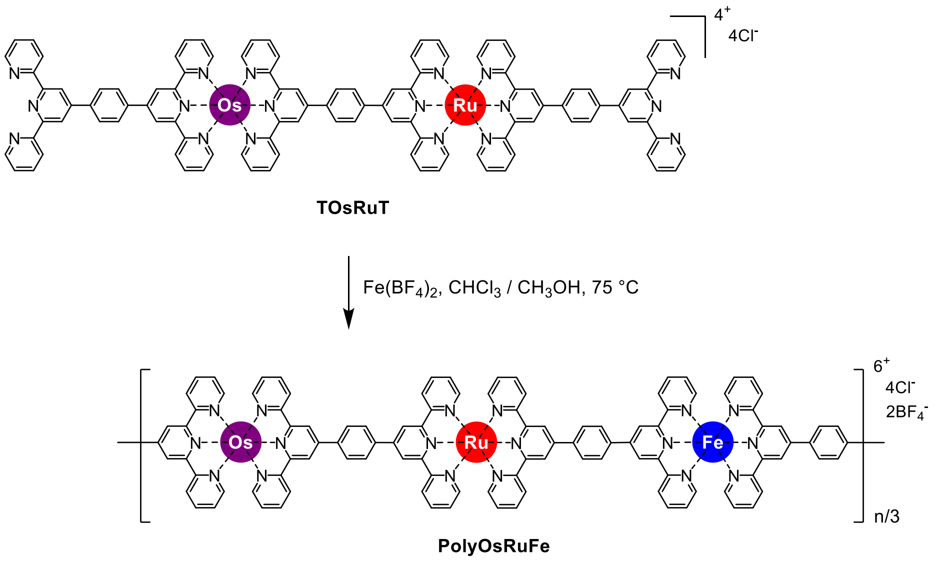

In a 25 mL round-bottom flask TRuOsT (25 mg, 12.1 μmol) was dissolved in 6 mL $CHCl_3$ and 4 mL CH_3OH followed by addition of and $Fe(BF_4)_2$ $6H_2O$ (4.05 mg, 12.1 μmol) in 2 mL MeOH under stirring. The reaction mixture was heated at 75 °C for 24 h and cooled to room temperature. The precipitate was filtered off and washed by CH_3OH, CH_3OH:$CHCl_3$ (1:1, v/v) and again CH_3OH. The residue was dried under vacuum to give polyOsRuFe as deep purple color solid (26.4 mg, 90% yield). **^{1}H NMR** (DMSO-d$_6$, 400 MHz, ppm) δ 9.91-9.65 (brm, 12H), 9.28-8.91 (brm, 24H), 8.14 (br, 12H), 7.72-7.36 (brm, 24H). **UV-Vis** [5×10^{-6} M in DMSO]: 311 nm (for π-π* transition), 502 nm [MLCT for <tpy-Ru(II)-tpy> connectivity + singlet MLCT for <tpy-Os(II)-tpy> connectivity], 575 nm [MLCT for <tpy-Fe(II)-tpy> connectivity], and 671 nm [triplet MLCT for <tpy-Os(II)-tpy> connectivity]. **CV** ($E_{1/2}$): 0.69, 0.86 and 1.01 V for Os(II)/Os(III), Fe(II)/Fe(III) and Ru(II)/Ru(III) redox pair, respectively.

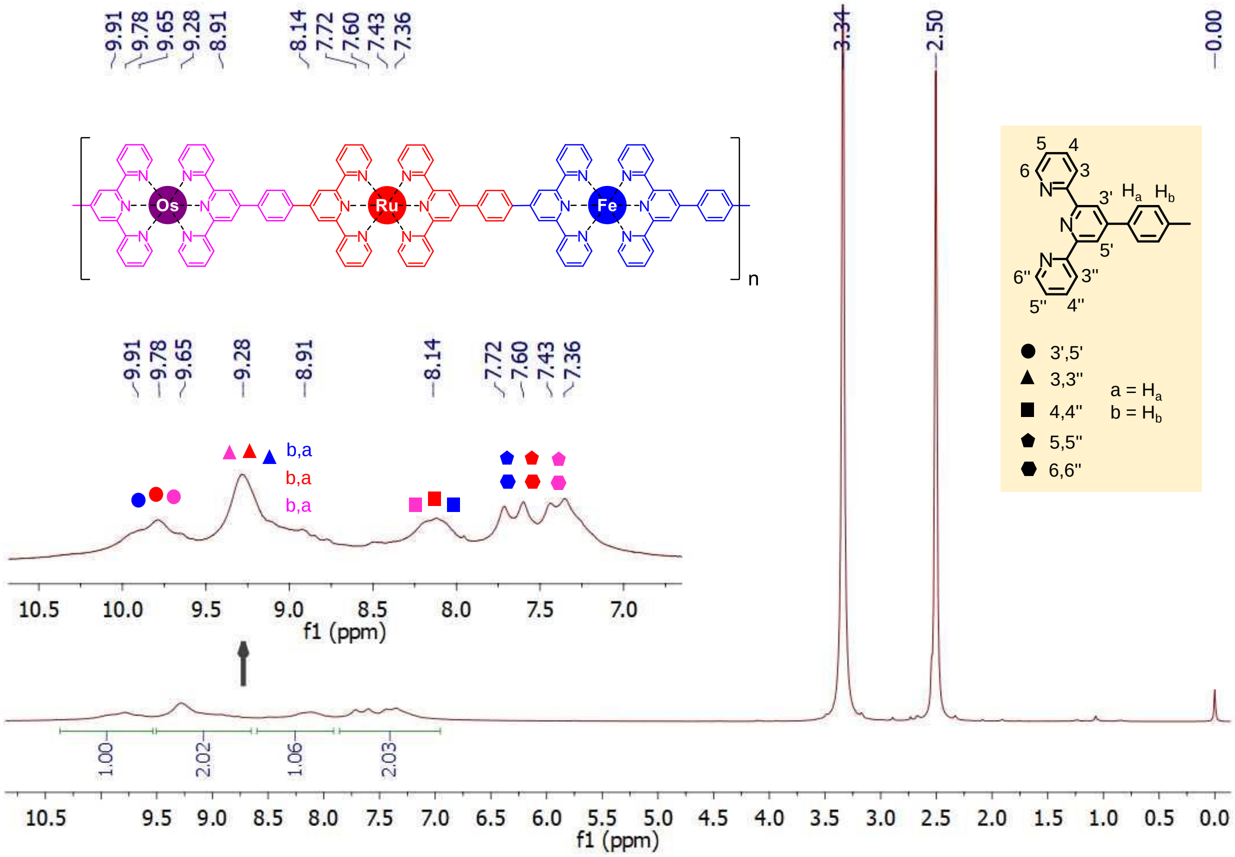

Supplementary Figure 19. ^{1}H NMR spectrum of polyOsRuFe in DMSO-d$_6$.

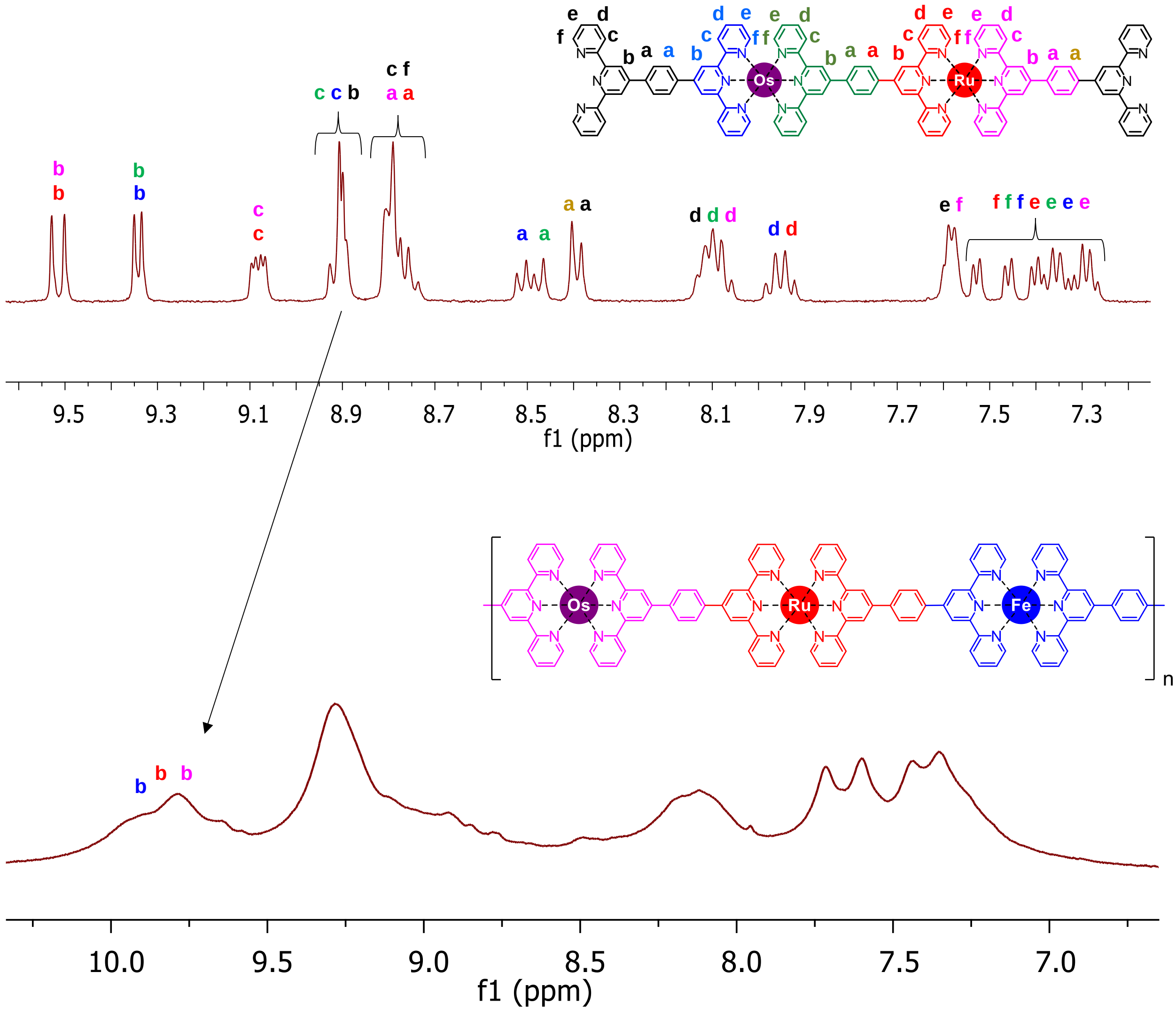

Supplementary Figure 20. Partial comparison of the ^{1}H NMR spectra of TOsRuT with polyOsRuFe to show 3',5' peak (denoted by 'b') shifting of free tpy units of TOsRuT in lower field upon complexation with Fe(II) (using arrow symbol). Assignment of proton: b = 3',5'; c = 3,3"; d = 4,4"; e = 5,5"; f = 6,6".

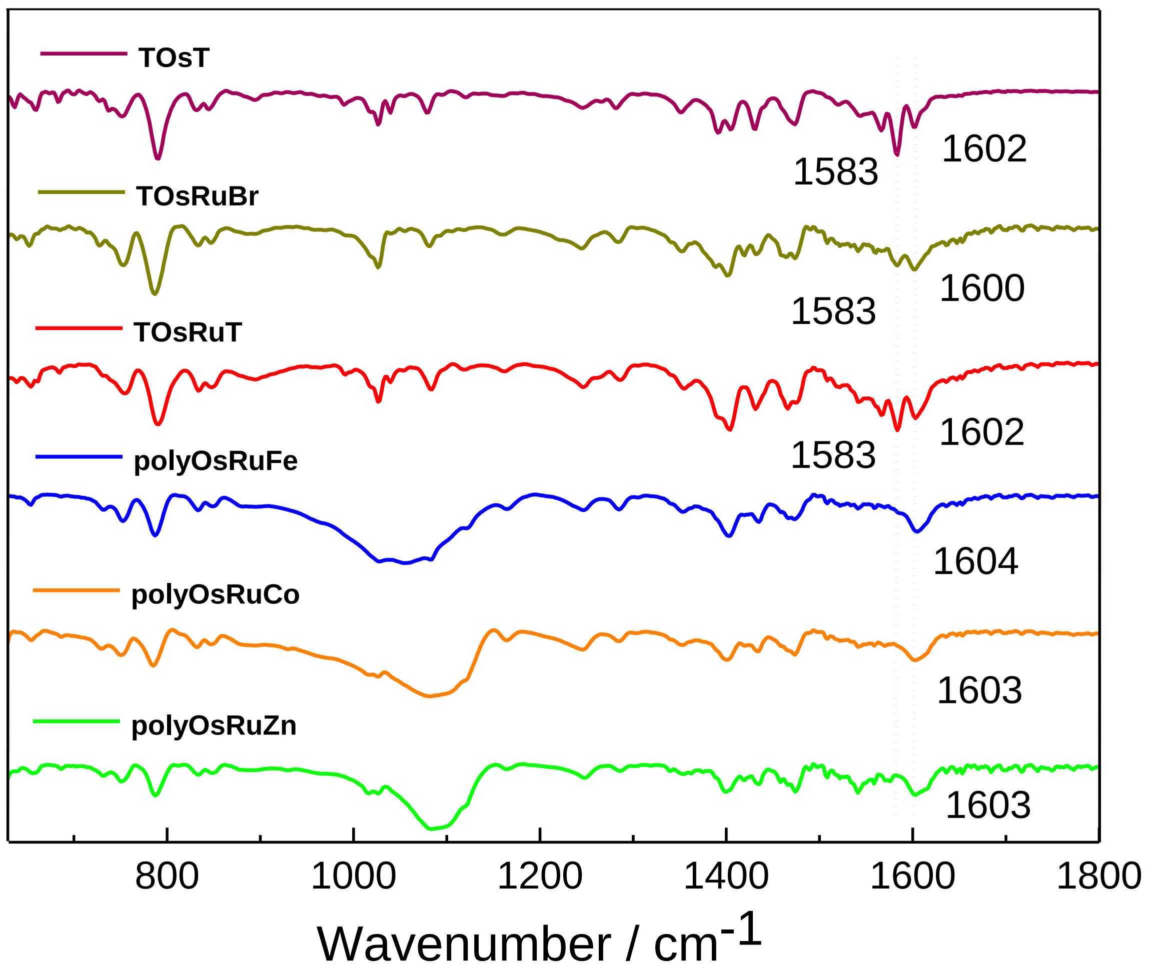

Supplementary Figure 21. FT-IR spectra of TOsT, TOsRuBr, TOsRuT, polyOsRuFe, polyOsRuCo, and polyOsRuZn.

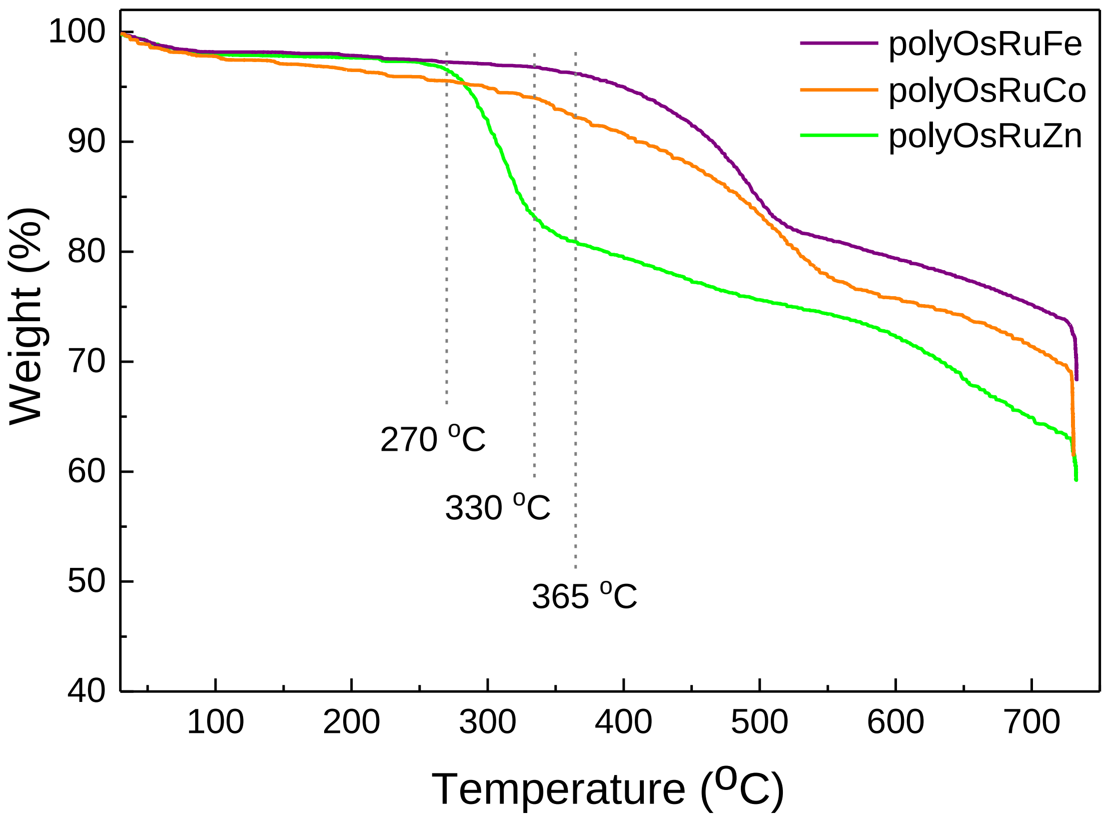

Supplementary Figure 22. TGA analysis of the HTMSPs under N_2 atmosphere.

The TGA analysis of HTMSPs exhibits two degradation points. First degradation is for breaking of the polymer chain at around 365 °C for polyOsRuFe, 330 °C for polyOsRuCo, and 270 °C for polyOsRuZn. The second degradation is for breaking of the ligand backbone at around 700°C, suggesting high thermal stability of the HTMSPs.

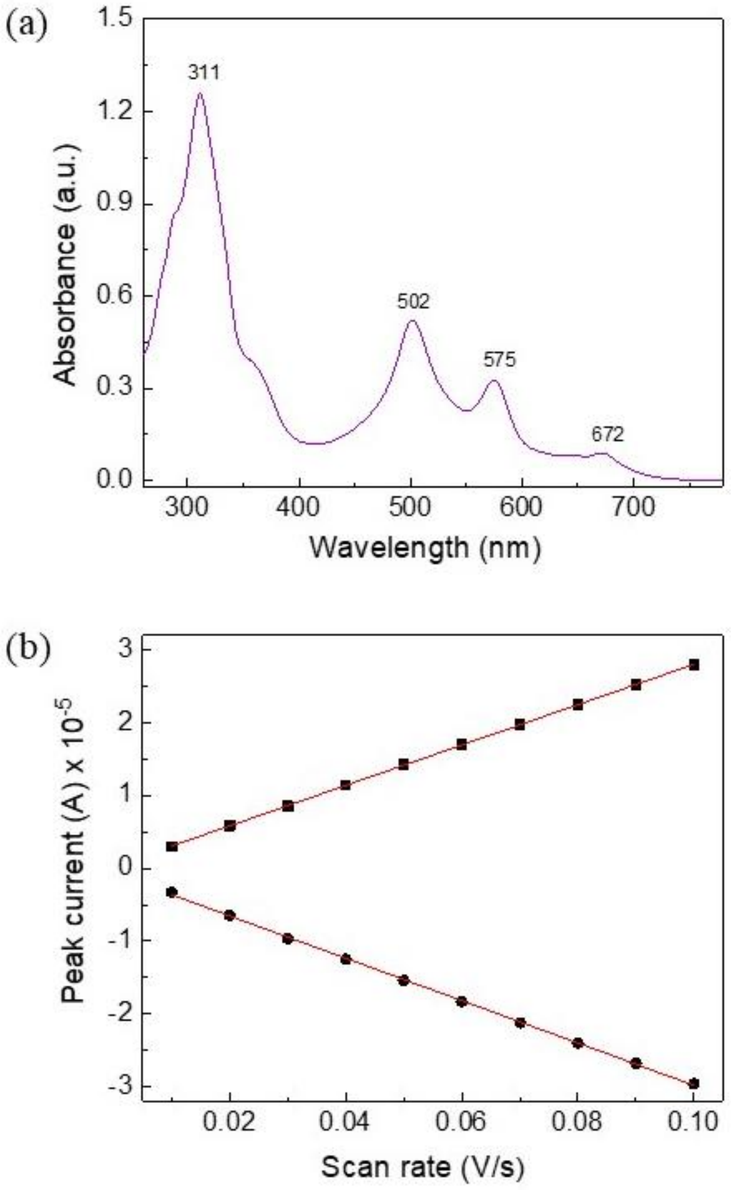

Supplementary Figure 23. a UV-vis spectrum (5 × 10^{-6} M in DMSO) and **b** Linear correlations between the peak current and the scan rate during oxidation (top) and reduction (bottom) processes (R^2 > 0.99 for fitting) of polyOsRuFe. Cyclic voltammograms of polyOsRuFe was measured with scan rates of 0.01-0.1 V/s in three-electrode system (glassy carbon as working electrode, platinum wire as counter electrode, and Ag/Ag^+ as reference electrode, electrolyte: 0.1 M $LiClO_4$ in CH_3CN). The polyOsRuFe was dissolved in DMSO and drop casted on the glassy carbon electrode.

Supplementary Table 1. UV-vis absorption of TOsT, TOsRuT, and HTMSPs.

**Compound**	λ_{ab} **(nm) for**			
	π-π* transition	MLCT for <tpy-Ru(II)-tpy> connectivity	Singlet and triplet MLCT for <tpy-Os(II)-tpy> connectivity	MLCT for <tpy-Fe(II)-tpy> connectivity
TOsT	314		493, 669	
TOsRuT	311	500	500, 671	
polyOsRuFe	311	502	502, 671	575
polyOsRuCo	309	499	499, 671	
polyOsRuZn	310	496	496, 670	

Supplementary Table 2. Electrochemical property of HTMSPs.

**Compound**	**Redox potential (**$E_{1/2}$**; V)**			
	Os(II)/Os(III)	Fe(II)/Fe(III)	Ru(II)/Ru(III)	Co(II)/Co(I)
polyOsRuFe	0.58	0.76	0.92	
polyOsRuCo	0.58		0.91	-1.29
polyOsRuZn	0.60		0.94	

Supplementary Table 3. Comparison of the observed redox potential of Os(II), Fe(II) and Ru(II) in polyOsRuFe with previously reported heterometallic complexes and polymers containing identical coordinating ligand.

**Supramolecular systems (discrete complexes/molecules and polymers) containing identical polypyridyl ligands and Os(II)Ru(II)Fe(II) / Os(II)Ru(II) / Os(II)Fe(II) / Ru(II)Fe(II) system**	**Cyclic voltammetry behavior (scan rate, electrolyte)**	**Redox potential ($E_{1/2}$; V) For Os(II)/Os(III)**	**Redox potential ($E_{1/2}$; V) For Fe(II)/Fe(III)**	**Redox potential ($E_{1/2}$; V) For Ru(II)/Ru(III)**	**Reference**
Ru(II)-Fe(II) containing polymer	Two reversible redox waves (50 mV/s, 0.1 M $LiClO_4$ in CH_3CN, *vs.* Ag/Ag^+)		0.77	0.93	*Molecules* **25**, 5261 (2020)
Os(II)-Fe(II) containing polymer	Two reversible redox waves (50 mV/s, 0.1 M $LiClO_4$ in CH_3CN, *vs.* Ag/Ag^+)	0.55	0.72		*Macromol. Rapid Commun.* **41**, 1900384 (2020)
Ru(II)-Os(II) dinuclear complex	Two reversible redox waves (*vs.* SEC)	0.95		1.39	*J. Chem. Soc., Faraday Trans.* **92**, 2223-2238 (1996)
Ru(II)-Os(II) complex	Two reversible redox waves (50 mV/s, 0.1 M TBAP in DMF, *vs.* Fc/Fc^+)	0.56		0.90	*Chem. Eur. J.* **8**, 130-136 (2002)
Os(II)-Ru(II)-Fe(II) containing polymer (polyOsRuFe)	Three reversible redox waves (100 mV/s, 0.1 M $LiClO_4$ in CH_3CN, *vs.* Ag/Ag^+)	0.58	0.76	0.92	This work

1.5. Synthesis and characterization of polyOsRuCo

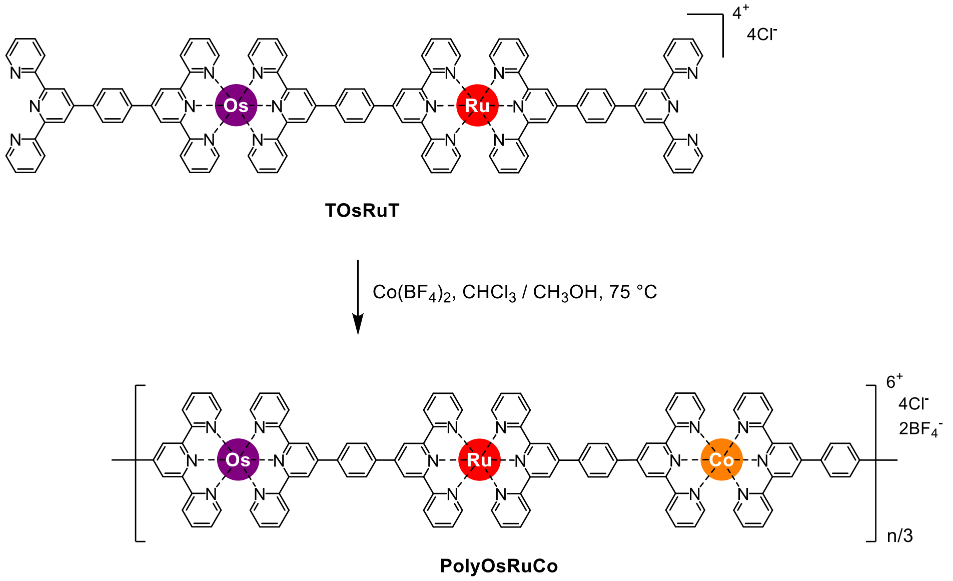

The preparation and purification polyOsRuCo was done following the same procedure as described for polyOsRuFe using $Co(BF_4)_2$ $6H_2O$ as the metal salt. The polyOsRuCo was obtained as black solid (88% yield). **^{1}H NMR** (DMSO-d_6, 400 MHz, ppm) δ 10.85-10.55 (brm, 6H), 9.96-8.96 (brm, 30H), 8.40-7.35 (brm, 36H). **UV-Vis** [5×10^{-6} M in DMSO]: 309 nm (for π-π* transition), 499 nm [MLCT for <tpy-Ru(II)-tpy> connectivity + singlet MLCT for <tpy-Os(II)-tpy> connectivity], and 671 nm [triplet MLCT for <tpy-Os(II)-tpy> connectivity]. **CV** ($E_{1/2}$): 0.58, 0.91 and -1.29 V for Os(II)/Os(III), Ru(II)/Ru(III) and Co(II)/Co(I) redox pair, respectively.

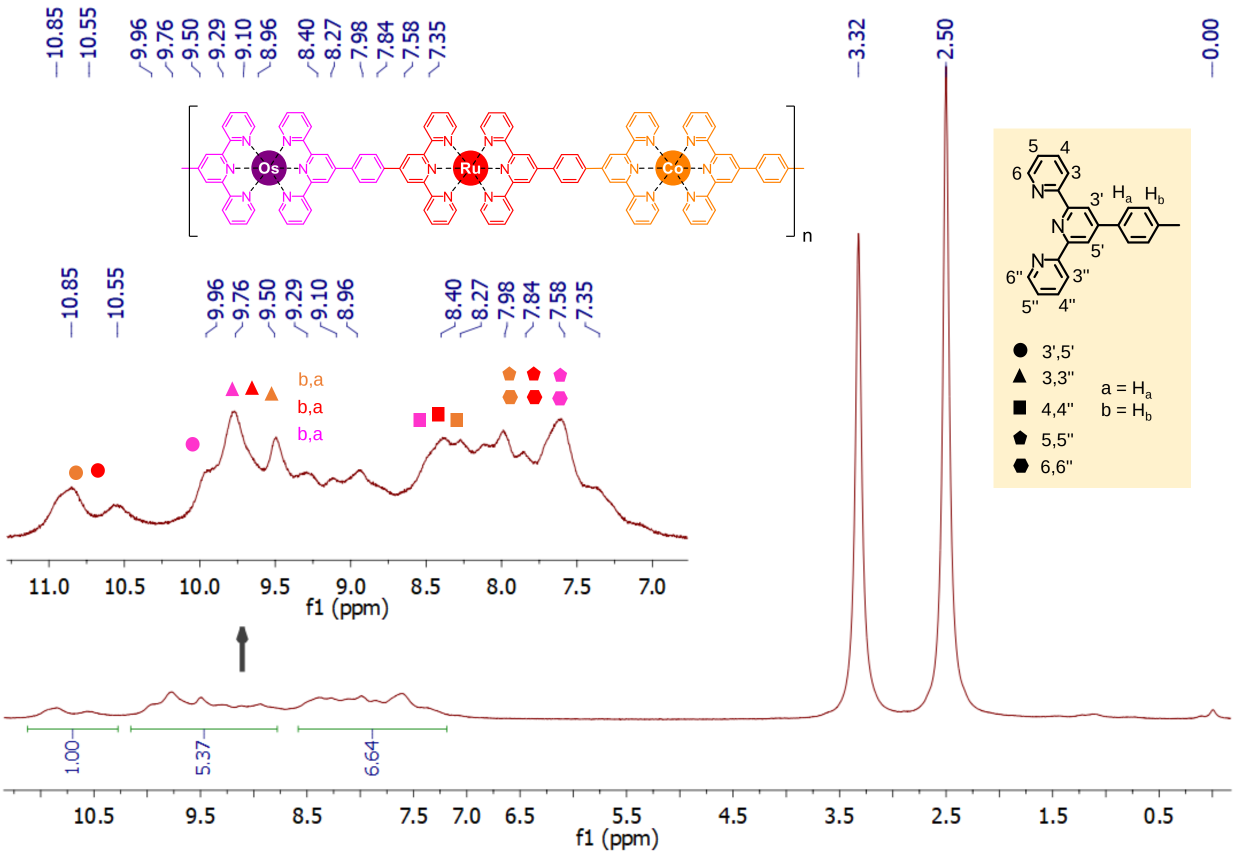

Supplementary Figure 24. ^{1}H NMR spectrum of polyOsRuCo in DMSO-d_6.

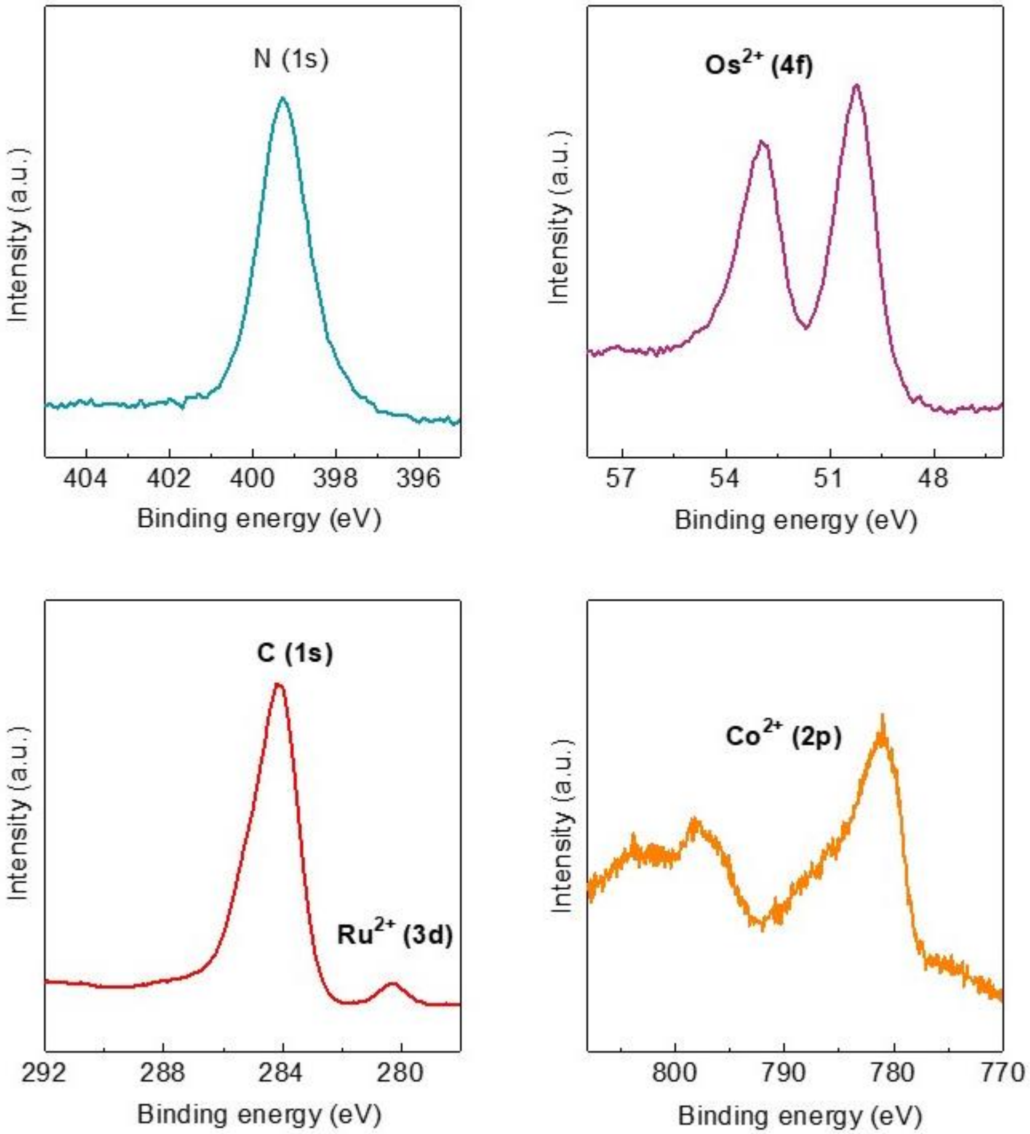

Supplementary Figure 25. Normalized X-ray photoelectron spectra of polyOsRuCo showing N (1s), Os^{2+} (4f), Ru^{2+} (3d), and Co^{2+} (2p) bands.

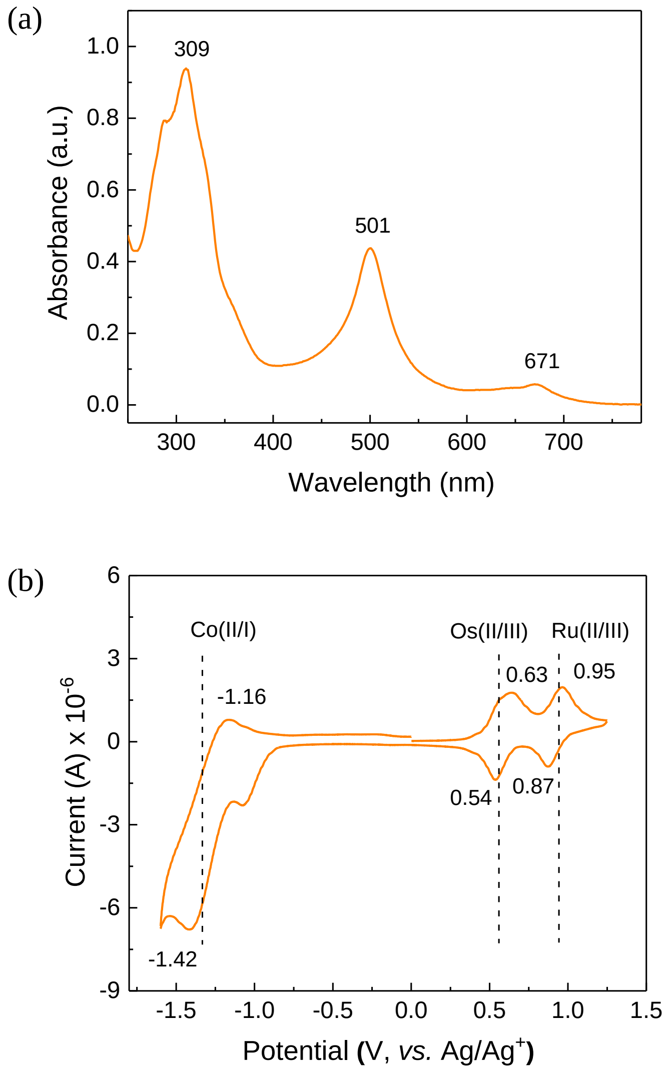

Supplementary Figure 26. a UV-vis spectrum (5 × 10^{-6} M in DMSO) and **b** cyclic voltammogram (in three electrode system; glassy carbon as working electrode, platinum wire as counter electrode, and Ag/Ag^+ as reference electrode, electrolyte: 0.1 M TBAP in acetone, scan rate 50 mV/s) of polyOsRuCo. The polyOsRuCo was dissolved in DMSO and drop casted on the glassy carbon electrode.

1.6. Synthesis and characterization of polyOsRuZn

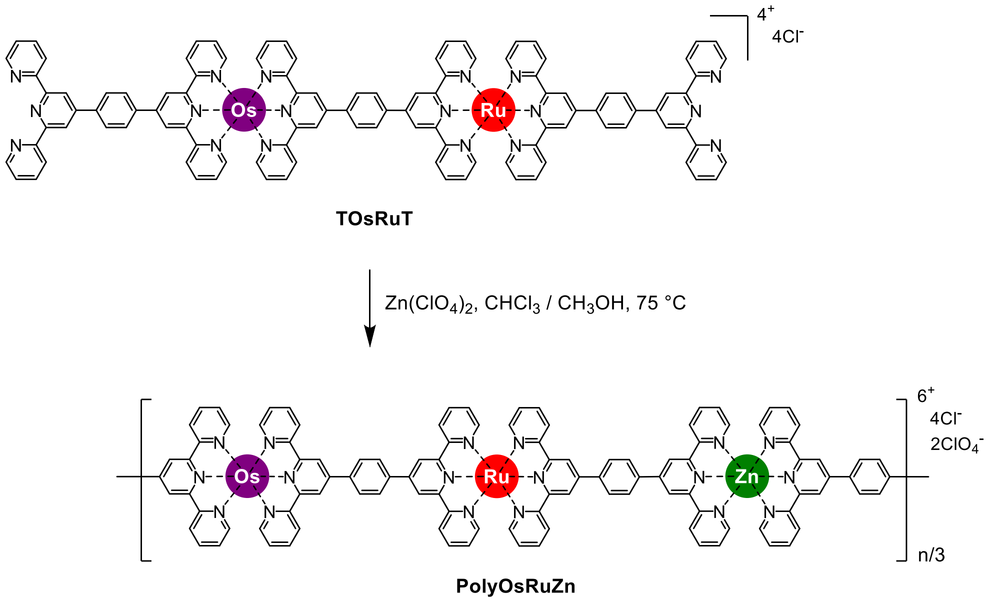

The preparation and purification polyOsRuZn was done following the same procedure as described for polyOsRuFe using $Zn(ClO_4)_2$ $6H_2O$ as the metal salt. The polyFeOsZn was obtained as black solid (79% yield). **^{1}H NMR** (DMSO-d_6, 400 MHz, ppm) δ 9.75 (br, 8H), 9.40-9.15 (brm, 16H), 8.94-8.77 (brm, 16H), 8.48 (br, 4H), 8.19-8.04 (brm, 12H), 7.72-7.57 (br, 8H) 7.41-7.32 (br, 8H). **UV-Vis** [5×10^{-6} M in DMSO]: 310 nm (for π-π* transition), 496 nm [MLCT for <tpy-Ru(II)-tpy> connectivity + singlet MLCT for <tpy-Os(II)-tpy> connectivity], and 670 nm [triplet MLCT for <tpy-Os(II)-tpy> connectivity]. **CV** ($E_{1/2}$): 0.60 and 0.94 V for Os(II)/Os(III), Ru(II)/Ru(III) redox pair, respectively.

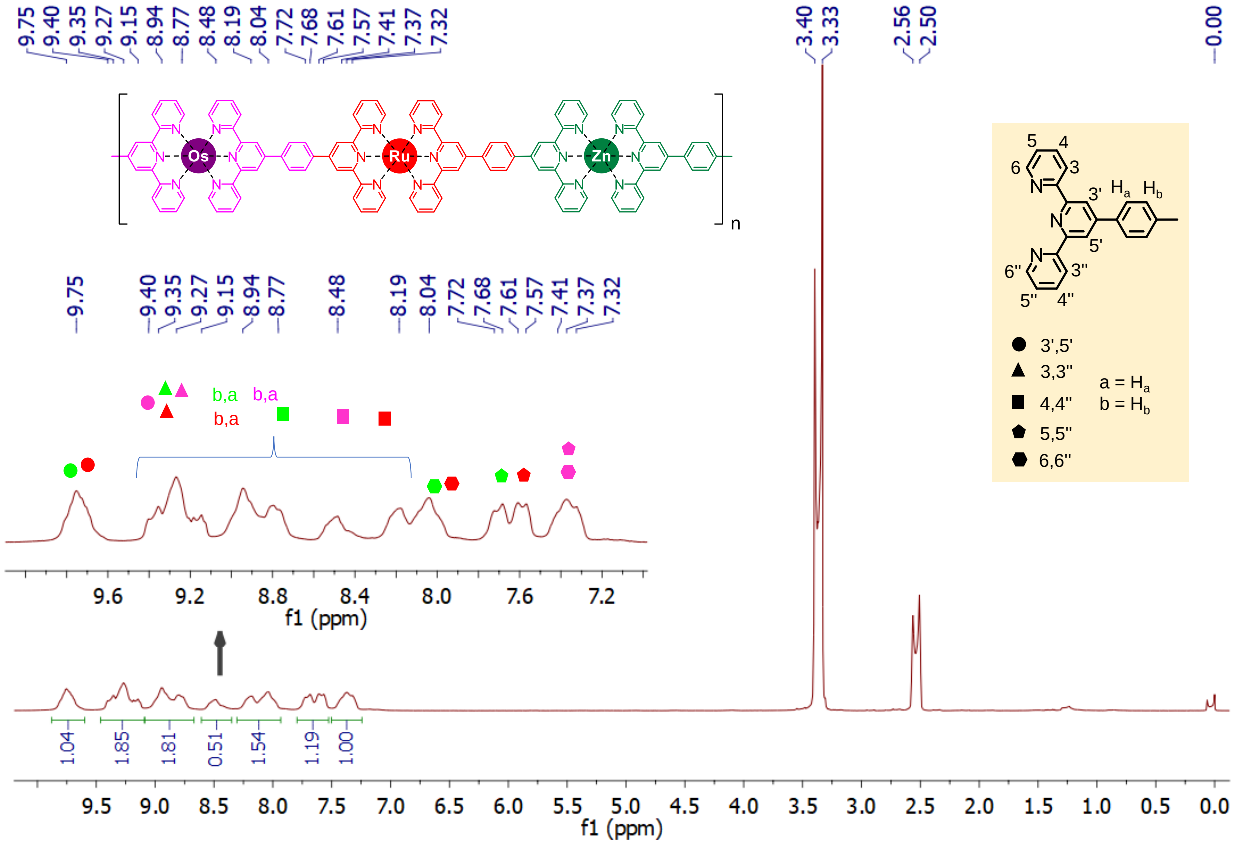

Supplementary Figure 27. ^1H NMR spectrum of polyOsRuZn in DMSO-d_6.

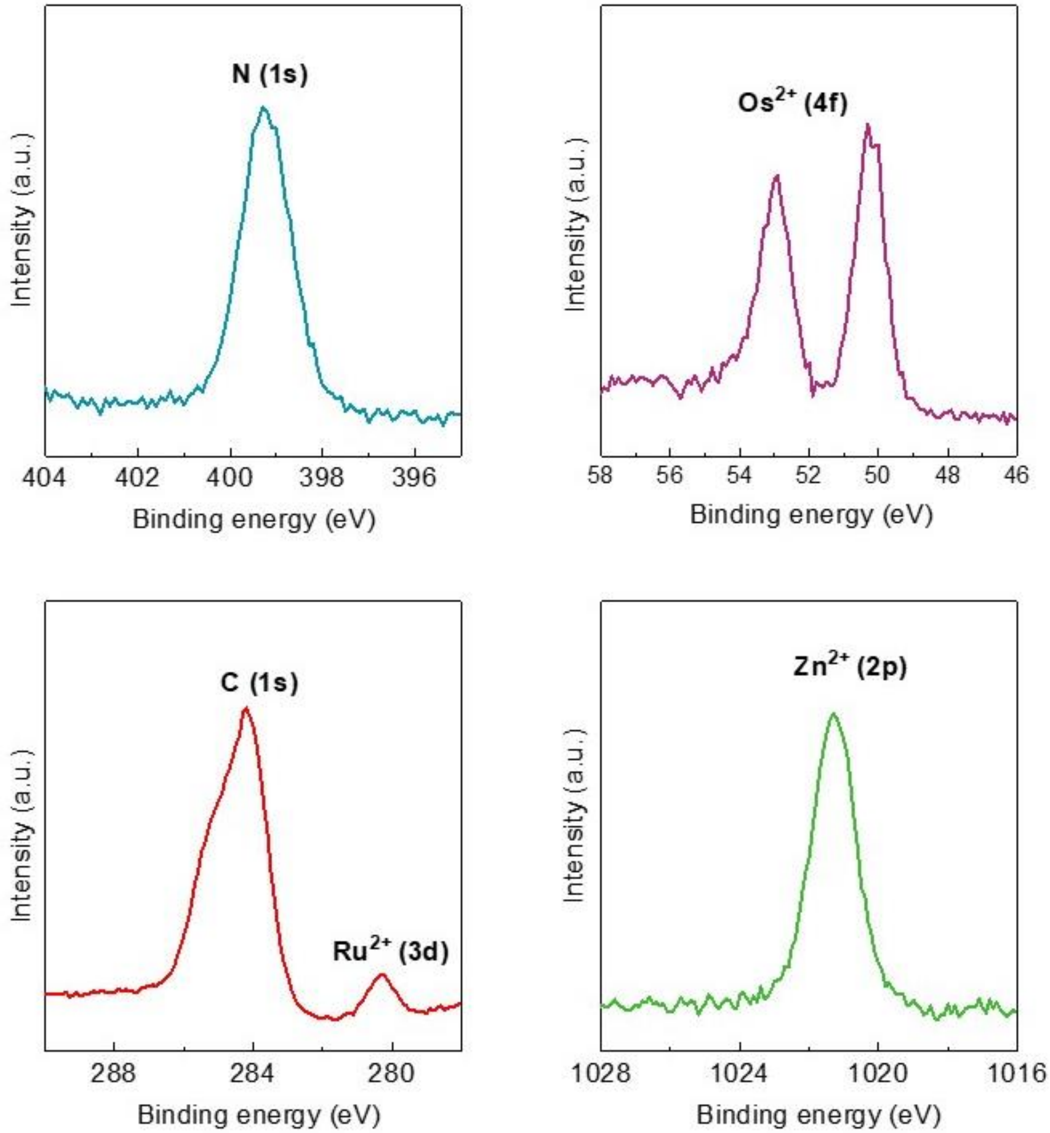

Supplementary Figure 28. Normalized X-ray photoelectron spectra of polyOsRuZn showing N (1s), Os^{2+} (4f), Ru^{2+} (3d), and Zn^{2+} (2p) bands.

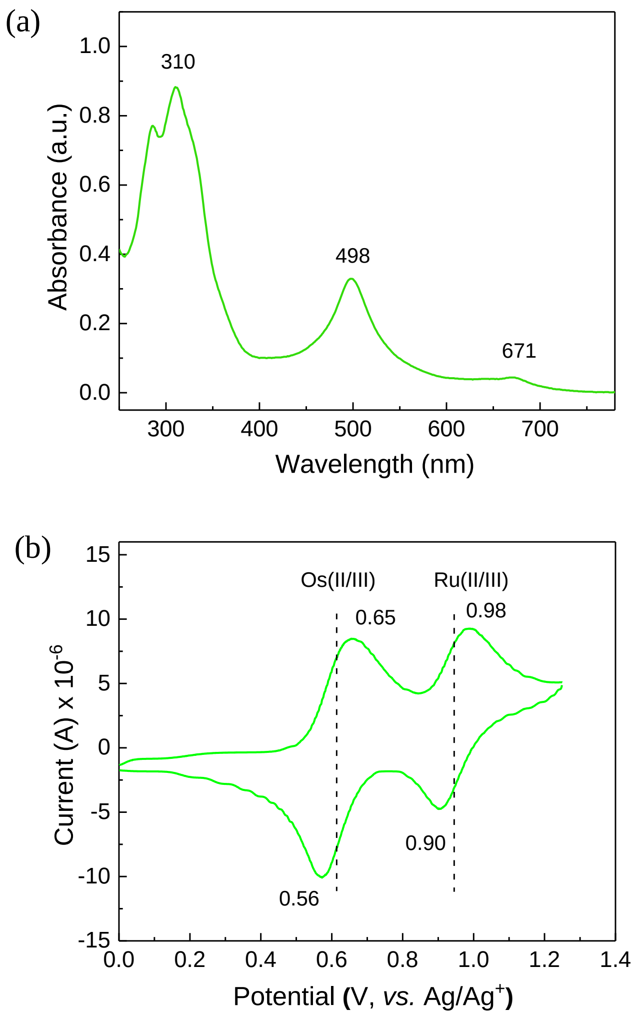

Supplementary Figure 29. a UV-vis spectrum (5×10^{-6} M in DMSO) and **b** cyclic voltammogram (in three electrode system; glassy carbon as working electrode, platinum wire as counter electrode, and Ag/Ag^{+} as reference electrode, electrolyte: 0.1 M $LiClO_4$ in CH_3CN, scan rate 50 mV/s) of polyOsRuZn. The polyOsRuZn was dissolved in DMSO and drop casted on the glassy carbon electrode.

1.7. Synthesis and characterization of polyOsRuFe-A (A: BF_4, Cl, PF_6, OAc)

Synthesis of polyOsRuFe-BF_4

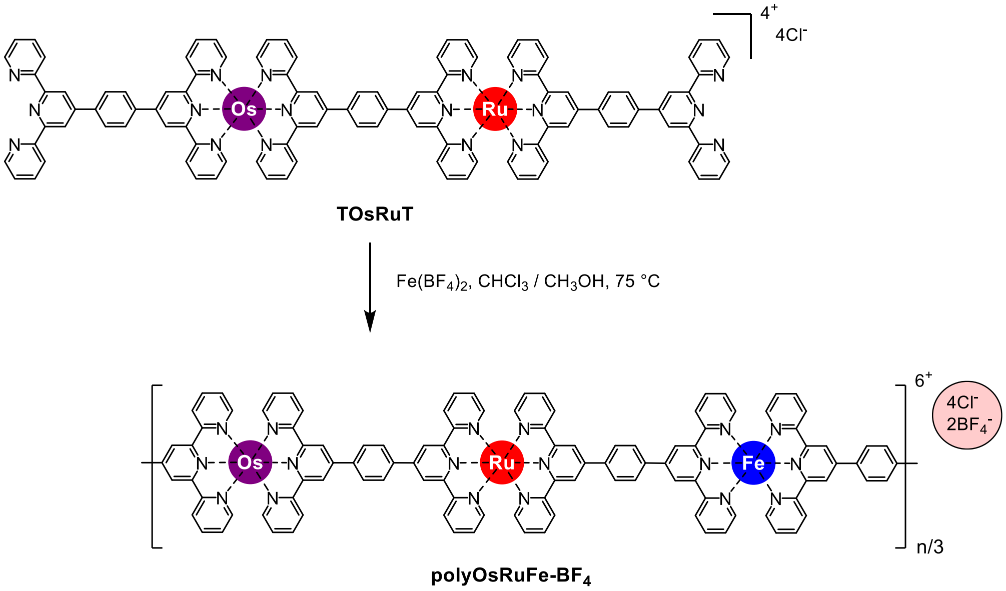

Synthesis of polyOsRuFe-BF_4 has been described above (see synthesis of polyOsRuFe).

Synthesis of polyOsRuFe-Cl

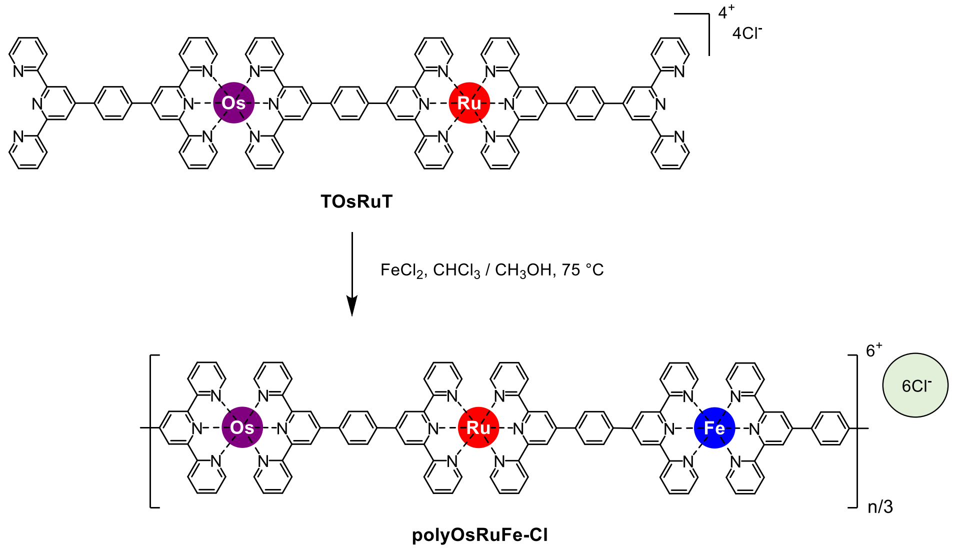

In a 25 mL round-bottom flask TRuOsT (25 mg, 12.1 µmol) was dissolved in 6 mL $CHCl_3$ and 4 mL CH_3OH followed by addition of and $FeCl_2$ $4H_2O$ (2.40 mg, 12.1 µmol) in 2 mL MeOH under stirring. The reaction mixture was heated at 75 °C for 24 h and cooled to room temperature. The precipitate was filtered off and washed by CH_3OH, CH_3OH:$CHCl_3$ (1:1, v/v) and again CH_3OH. The residue was dried under vacuum to give polyOsRuFe-Cl as deep purple color solid (25.3 mg, 92% yield). **UV-Vis** [5×10^{-6} M in DMSO]: 317 nm (for π-π* transition), 512 nm [MLCT for <tpy-Ru(II)-tpy> connectivity + singlet MLCT for <tpy-Os(II)-tpy> connectivity], 587 nm [MLCT for <tpy-Fe(II)-tpy> connectivity], and 682 nm [triplet MLCT for <tpy-Os(II)-tpy> connectivity]. **CV** ($E_{1/2}$): 0.59, 0.76 and 0.91 V for Os(II)/Os(III), Fe(II)/Fe(III) and Ru(II)/Ru(III) redox pair, respectively.

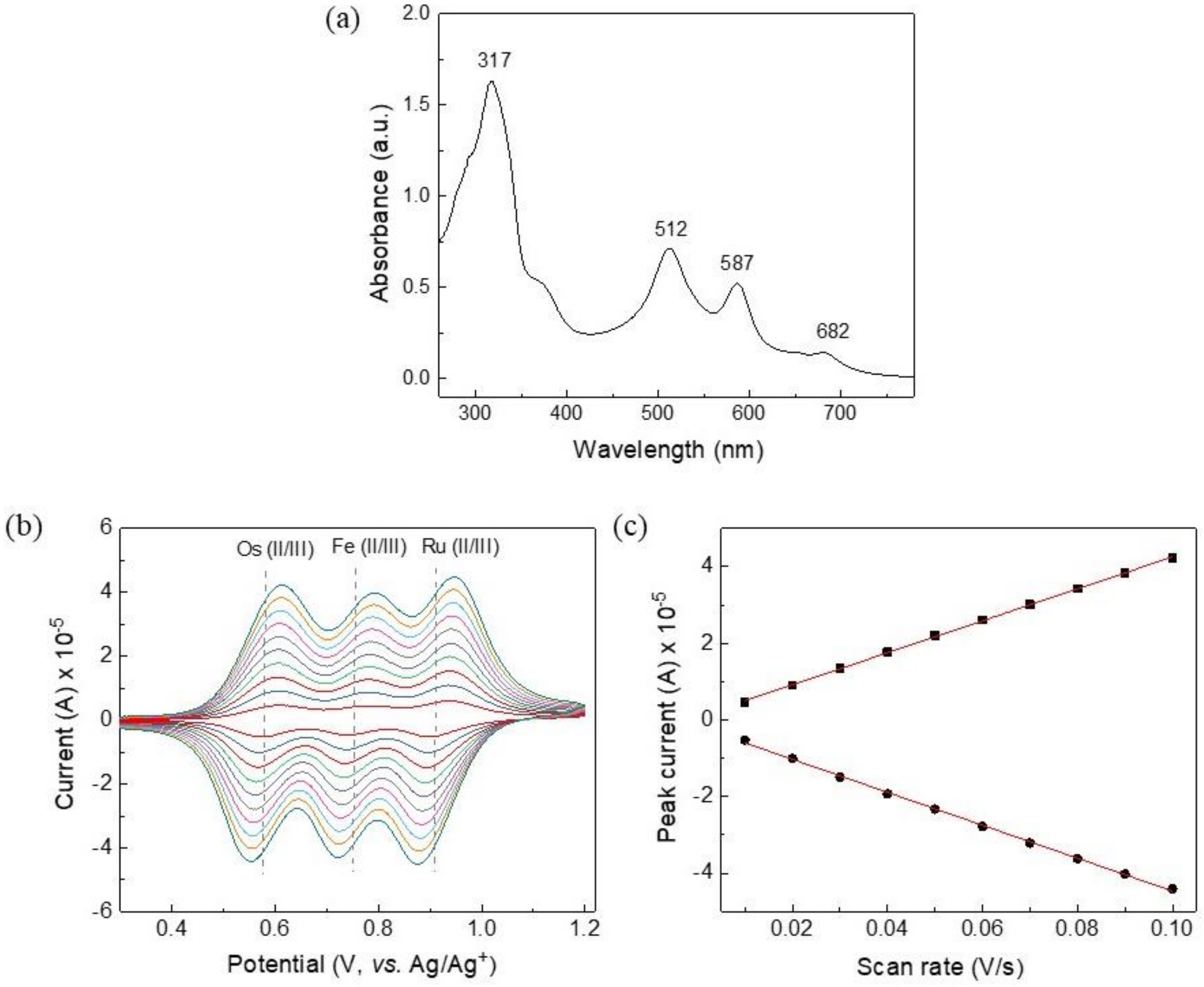

Supplementary Figure 30. a UV-vis spectrum (5×10^{-6} M in DMSO), **b** Cyclic voltammograms with scan rates of 0.01-0.1 V/s in three-electrode system (glassy carbon as working electrode, platinum wire as counter electrode, and Ag/Ag^+ as reference electrode, electrolyte: 0.1 M $LiClO_4$ in CH_3CN) and **c** Linear correlations between the peak current and the scan rate during oxidation (top) and reduction (bottom) processes (R^2 > 0.99 for fitting) of polyOsRuFe-Cl. The polyOsRuFe-Cl was dissolved in DMSO and drop casted on the glassy carbon electrode.

Synthesis of polyOsRuFe-PF_6

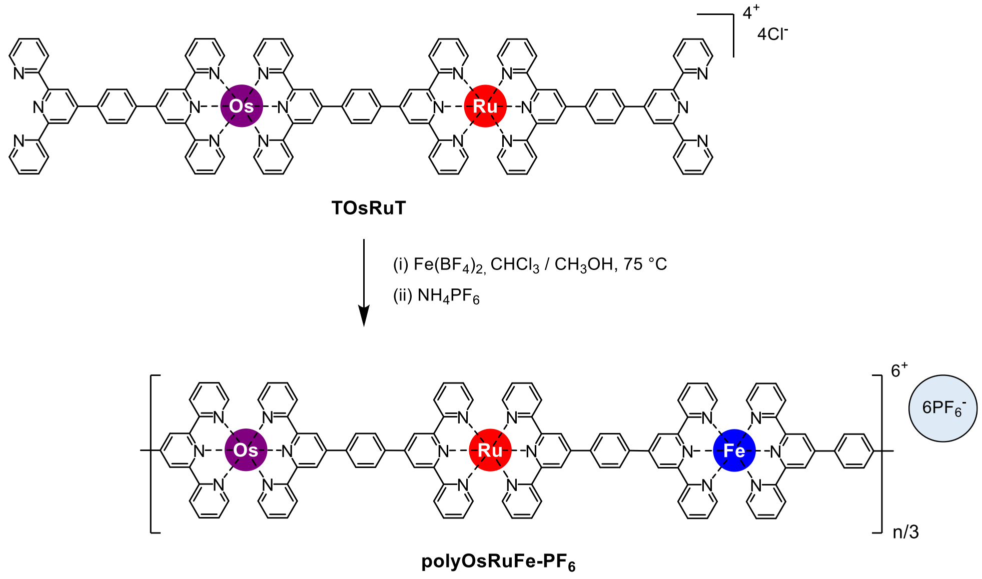

The polyOsRuFe-PF_6 was prepared from polyOsRuFe-BF_4. In a 50 mL beaker, polyOsRuFe-BF_4 (30 mg) was dissolve in 8 mL DMSO followed by addition of saturated aqueous solution of NH_4PF_6. After that a precipitate is formed, which was filtered and washed with CH_3OH and diethyl ether followed by drying under vacuum to afford polyOsRuFe-PF_6 as deep purple color solid with almost quantitative yield (29.5 mg; 98%). **UV-Vis** [5×10^{-6} M in CH_3CN]: 310 nm (for π-π* transition), 501 nm [MLCT for <tpy-Ru(II)-tpy> connectivity + singlet MLCT for <tpy-Os(II)-tpy> connectivity], 572 nm [MLCT for <tpy-Fe(II)-tpy> connectivity], and 671 nm [triplet MLCT for <tpy-Os(II)-tpy> connectivity]. **CV** ($E_{1/2}$): 0.57, 0.74 and 0.90 V for Os(II)/Os(III), Fe(II)/Fe(III) and Ru(II)/Ru(III) redox pair, respectively.

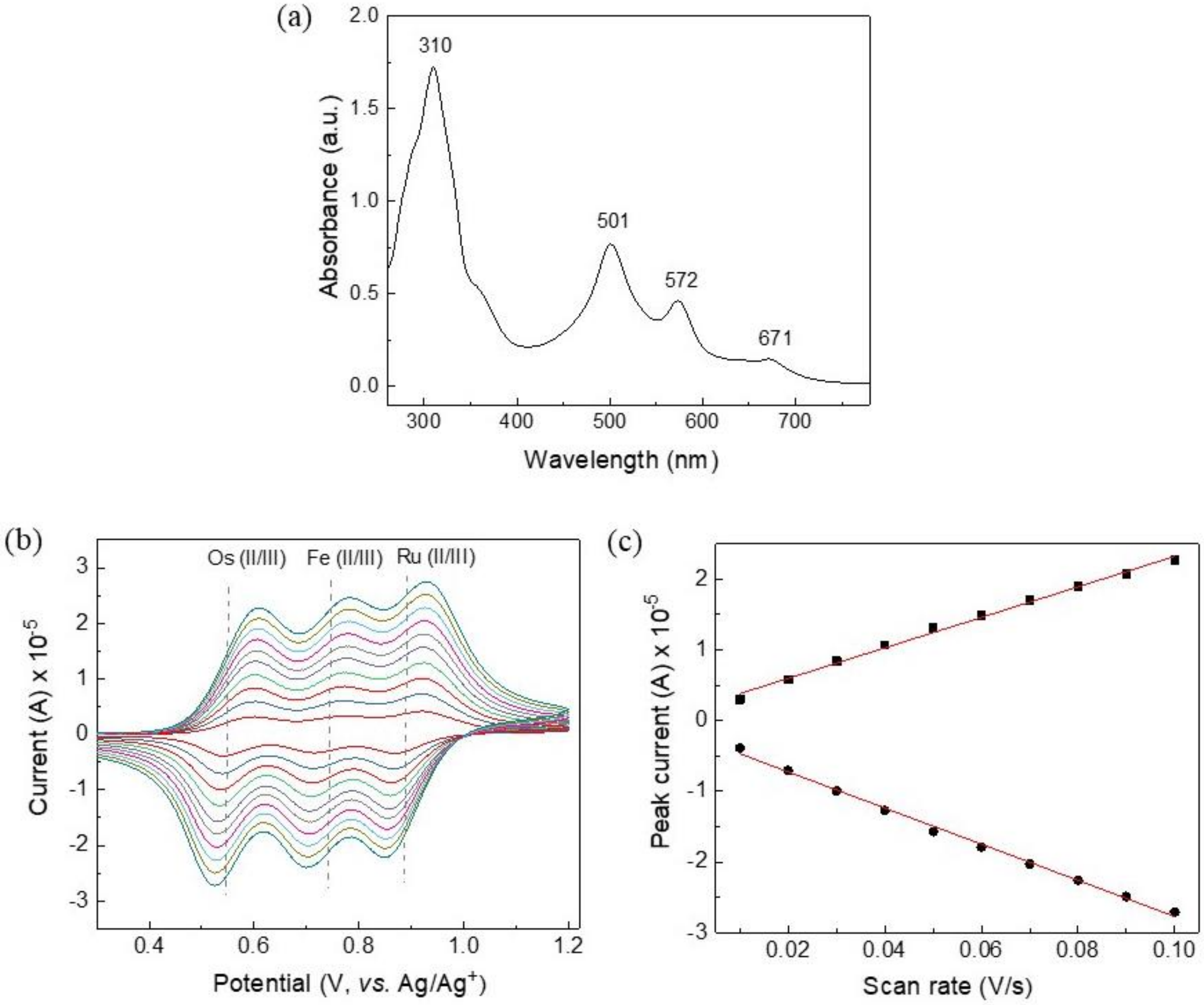

Supplementary Figure 31. a UV-vis spectrum (5×10^{-6} M in CH_3CN), **b** Cyclic voltammograms with scan rates of 0.01-0.1 V/s in three-electrode system (glassy carbon as working electrode, platinum wire as counter electrode, and Ag/Ag^+ as reference electrode, electrolyte: 0.1 M TBAP in acetone) and c Linear correlations between the peak current and the scan rate during oxidation (top) and reduction (bottom) processes (R^2 > 0.99 for fitting) of polyOsRuFe-PF_6. The polyOsRuFe-PF_6 was dissolved in CH_3CN and drop casted on the glassy carbon electrode.

Synthesis of polyOsRuFe-OAc

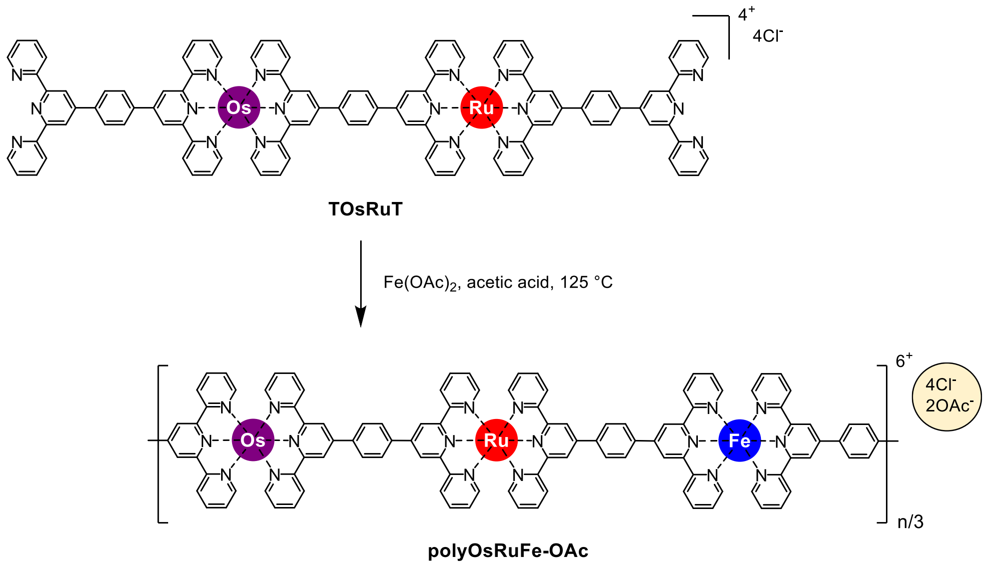

In a 25 mL round-bottom flask TRuOsT (50 mg, 24.2 µmol) was dissolved in 15 mL acetic acid. Then, solid $Fe(OAc)_2$ (4.20 mg, 24.2 µmol) was added to it under stirring and refluxed for 24 h at 125 °C. The reaction mixture was cooled to room temperature, filtered to remove insoluble residue, and acetic acid was evaporated to get polyOsRuFe-OAc as deep purple color solid (38.2 mg, 70% yield). **UV-Vis** [5×10^{-6} M in CH_3OH]: 310 nm (for π-π* transition), 501 nm [MLCT for <tpy-Ru(II)-tpy> connectivity + singlet MLCT for <tpy-Os(II)-tpy> connectivity], 573 nm [MLCT for <tpy-Fe(II)-tpy> connectivity], and 671 nm [triplet MLCT for <tpy-Os(II)-tpy> connectivity]. **CV** ($E_{1/2}$): 0.69, 0.86 and 1.01 V for Os(II)/Os(III), Fe(II)/Fe(III) and Ru(II)/Ru(III) redox pair, respectively.

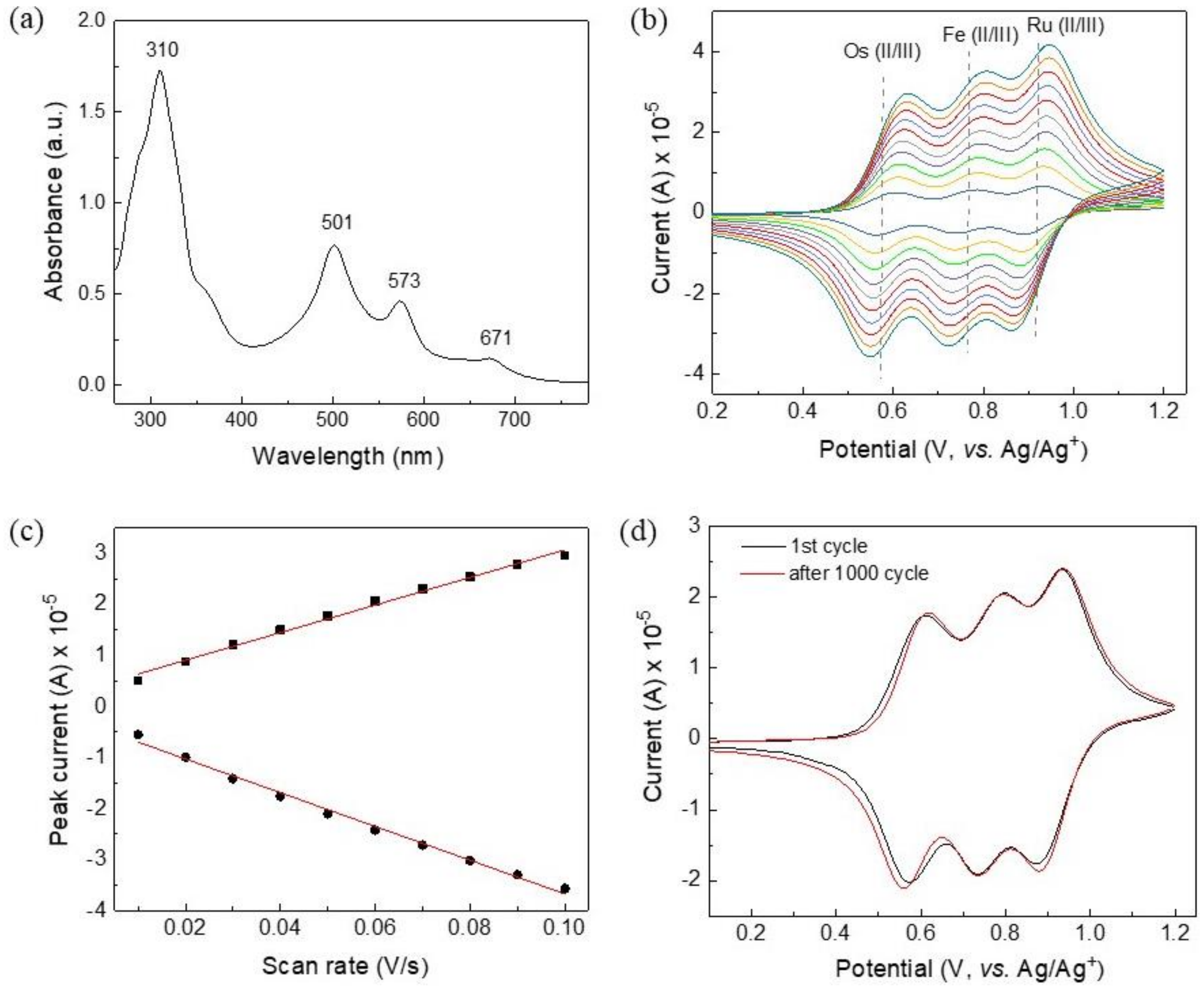

Supplementary Figure 32. a UV-vis spectrum (5×10^{-6} M in CH_3OH), **b** Cyclic voltammograms with scan rates of 0.01-0.1 V/s in three-electrode system (glassy carbon as working electrode, platinum wire as counter electrode, and Ag/Ag^+ as reference electrode, electrolyte: 0.1 M $LiClO_4$ in CH_3CN), **c** Linear correlations between the peak current and the scan rate during oxidation (top) and reduction (bottom) processes ($R^2 > 0.99$ for fitting) of polyOsRuFe-OAc, and **d** Cyclic voltammograms of polyOsRuFe-OAc for1st cycle and after 1000 cycles at a scan rate of 50 mV/s. The polyOsRuFe-OAc was dissolved in CH_3OH and drop casted on the glassy carbon electrode.

Supplementary Table 4. UV-vis absorption of polyOsRuFe-A; where A = Cl, PF_6, OAc.

**Compound**	**λ_{ab} (nm) for**			
	π-π* transition	MLCT for <tpy-Ru(II)-tpy> connectivity	Singlet and triplet MLCT for <tpy-Os(II)-tpy> connectivity	MLCT for <tpy-Fe(II)-tpy> connectivity
polyOsRuFe-Cl	317	512	512, 682	587
polyOsRuFe-PF_6	310	501	501, 671	572
polyOsRuFe-OAc	310	501	501, 671	573

Supplementary Table 5. Electrochemical property of polyOsRuFe-A; where A = Cl, PF_6, OAc.

**Compound**	**Redox potential ($E_{1/2}$; V)**		
	Os(II)/Os(III)	Fe(II)/Fe(III)	Ru(II)/Ru(III)
polyOsRuFe-Cl	0.58	0.76	0.92
polyOsRuFe-PF_6	0.57	0.74	0.90
polyOsRuFe-OAc	0.59	0.75	0.90

Supplementary Table 6. Solubility of the polyOsRuFe-A (A: BF_4, Cl, PF_6, OAc) in different solvents.[a]

**polyOsRuFe-A** *	$CHCl_3$ CH_2Cl_2	DMF	DMSO	CH_3CN	CH_3OH	EtOH	H_2O
polyOsRuFe-BF_4	X	√	√	X	X	X	X
polyOsRuFe-Cl	X	√	√	X	X	X	X
polyOsRuFe-PF_6	X	√	√	√ #	X	X	X
polyOsRuFe-OAc	X	√	√	X	√ ϕ	√ ο	√ †

[a] The solubility of polyOsRuFe-A (A: BF_4, Cl, PF_6, OAc) was examined by taking 1mg polymer in 4mL solvent (1.3×10^{-4} M) at 25 °C [√ = soluble, × = insoluble]. # The maximum solubility was observed up to 6.0×10^{-5} M. ϕο† The maximum solubility of polyOsRuFe-OAc was observed up to 2.0×10^{-3} M in MeOH, 1.1×10^{-3} M in EtOH, and 9.6×10^{-4} M in H_2O, respectively. Polymer concentration was calculated with respect to molecular weight of the repeating unit.

1.8. Spectroelectrochemical study of polyOsRuFe-OAc film on ITO/glass

Preparation of a polymer film on ITO/glass: A film of polyOsRuFe-OAc was prepared on an ITO-coated glass substrate (ITO/glass) by spin-coating. A CH_3OH solution (3 mg/mL) of polyOsRuFe-OAc was spin-coated (at 120 rpm for 600 s) on ITO/glass and the prepared film was then dried at room temperature for another 20 min followed by removing a small coating in each side to make an active area of the film of 1 cm × 1 cm.

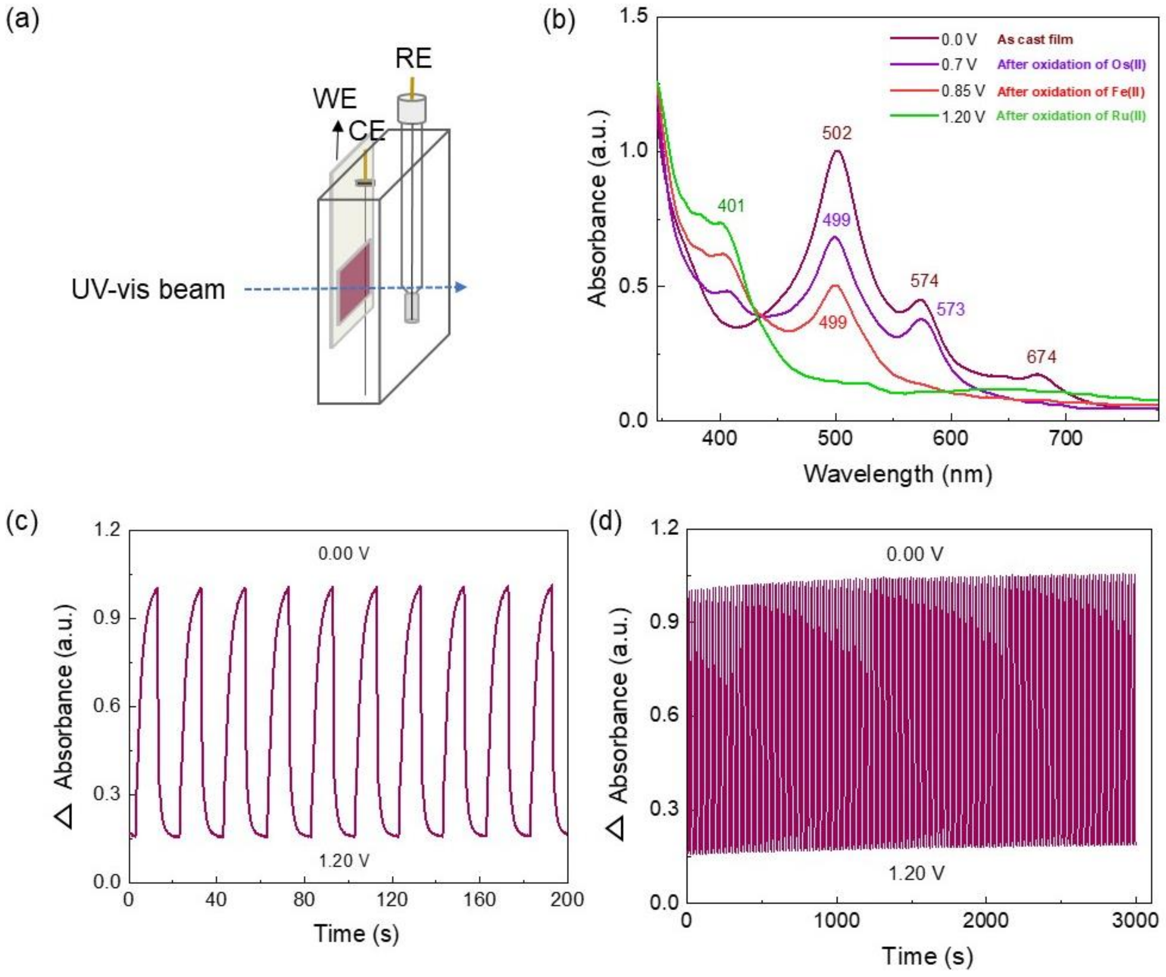

Supplementary Figure 33. a Schematic view of the three-electrode electrochemical cell with UV-vis beam path for *in situ* spectroelectrochemical measurement using 0.1 M $LiClO_4$ in CH_3CN as electrolyte **b** In situ UV-vis spectra of a polyOsRuFe-OAc film on ITO/glass at different applied potentials of 0.70 V, 0.85 V, and 1.20 V for stepwise oxidation of Os(II), Fe(II), and Ru(II), respectively **c** Change in absorbance of polyOsRuFe-OAc film on ITO/glass for few cycles, and **d** for 300 cycles; monitored at 502 nm upon switching the potential between 0.0 and 1.2 V during chronoamperometry measurement (interval time: 5 s).

2. Supplementary References

1. Sun, Q. *et al.* Bright NUV mechanofluorescence from a terpyridine-based pure organic crystal. *Chem. Commun.* **54**, 94-97 (2018).
2. Duncan, T. V., Ishizuka, T. & Therien, M. J. Molecular Engineering of Intensely Near-Infrared Absorbing Excited States in Highly Conjugated Oligo(porphinato)zinc−(Polypyridyl)metal(II) Supermolecules. *J. Am. Chem. Soc.* **129**, 9691-9703 (2007).
3. Bera, M. K., Ninomiya, Y., Yoshida, T. & Higuchi, M. Precise Synthesis of Alternate Fe(II)/Os(II)-Based Bimetallic Metallo-Supramolecular Polymer. *Macromol. Rapid Commun.* **41**, 1900384 (2020).
4. Barigelletti, F. *et al.* Rigid Rod-Like Dinuclear Ru(II)/Os(II) Terpyridine-Type Complexes. Electrochemical Behavior, Absorption Spectra, Luminescence Properties, and Electronic Energy Transfer through Phenylene Bridges. *J. Am. Chem. Soc.* **116**, 7692-7699 (1994).
5. Chakraborty, S. & Newkome, G. R. Terpyridine-based metallosupramolecular constructs: tailored monomers to precise 2D-motifs and 3D-metallocages. *Chem. Soc. Rev.* **47**, 3991-4016 (2018).
6. Ludlow III, J. M. *et al.* Group 8 Metallomacrocycles – Synthesis, Characterization, and Stability. *Eur. J. Inorg. Chem.* **2015**, 5662-5668 (2015).
